# Modelling the impact of historic landscape change on soil erosion and degradation

**DOI:** 10.1038/s41598-023-31334-z

**Published:** 2023-03-27

**Authors:** Filippo Brandolini, Tim C. Kinnaird, Aayush Srivastava, Sam Turner

**Affiliations:** 1 McCord Centre for Landscape, School of History, Classics and Archaeology, Newcastle University, Armstrong Building, Newcastle upon Tyne, NE1 7RU UK; 2 School of Earth and Environmental Sciences, University of St Andrews, St Andrews, KY16 9AX UK

**Keywords:** Sustainability, Environmental impact, Geomorphology

## Abstract

International policies and guidelines often highlight the divide between ‘nature’ and ‘heritage’ in landscape management, and the weakness of monodisciplinary approaches. This study argues that historic agricultural practices have played a key role in shaping today’s landscapes, creating a heritage which affords opportunities for more sustainable landscape management. The paper develops a new interdisciplinary approach with particular reference to soil loss and degradation over the long term. It presents innovative methods for assessing and modelling how pre-industrial agricultural features can mitigate soil erosion risk in response to current environmental conditions. Landscape archaeology data presented through Historic Landscape Characterisation are integrated in a GIS-RUSLE model to illustrate the impact of varying historic land-uses on soil erosion. The resulting analyses could be used to inform strategies for sustainable land resource planning.

## Introduction

Land degradation is a critical environmental issue worldwide. The latest projections on climate change indicate that increasingly severe storm intensity and runoff will induce greater soil losses by water erosion in the future than in the past decades^1,2^. The ‘FAO Global Symposium on Soil Erosion’^3^ underlined how soil degradation can be accelerated by human activities, with substantial implications for future land productivity, rural livelihoods and biodiversity. Land Use and Land Cover (LULC) dynamics are the major anthropogenic drivers of soil loss and degradation^4^. Agriculture represents the largest LULC type worldwide and developing more sustainable agricultural systems is an urgent global challenge^5^. Among the main conclusions from the FAO Symposium were calls for joint efforts from all stakeholders and for collaboration among researchers to deal with this complex emergency at various scales^3^. The FAO Symposium noted that pre-industrial farming practices often contributed to maintaining healthy soils while modern intensive agriculture and mechanisation have frequently induced significant erosion^3^. Archaeological sciences can contribute by exploring social and environmental interactions to examine the impact of different practices over long periods of time^6–8^, and considering what made different practices sustainable or unsustainable in terms of past soil management. From a heritage perspective, many cultural landscapes have been created through agricultural practices such as terrace farming, agroforestry and integrated crop-animal farming which trace their origins far back into the past^9–12^. Archaeological research into such practices may have the potential to inform more sustainable soil management in the future^13^ (noting that the definition of ‘sustainable’ in this paper corresponds to the one used by FAO^3^).

In European countries, historic landscape change has occurred with unprecedented intensity over the last 70 years^14–16^. For instance, in mountainous regions of the Mediterranean, the decline in the importance of agriculture has led to a significant reduction of activities in rural areas, with progressive depopulation of villages and changes to the characteristic historic features of landscapes^17^. Since the 1950s, the widespread application of mechanised agriculture has resulted in major transformations of the landscape by enlarging fields, removing their physical boundaries and replacing traditional multi-cropping practices with monoculture systems which have enabled farmers to increase efficiency, productivity and profit^18,19^. Nevertheless, recent environmental studies and policies have recommended maintaining cultural and archaeological landscape features such as intercropping, agroforestry and cross slope barriers (e.g. hedgerows, stone walls, earth banks) for their benefits to ecosystems^3,20,21^. Such boundaries are also key components of historic landscapes. Recent geochronological analysis has confirmed the antiquity of such features in case-studies across the Mediterranean^22^. Thus, their conservation and valorisation are necessary not only to mitigate the effect of land degradation but also to preserve historic character for future generations. The benefits of leveraging landscape ecology and heritage together have been increasingly recognised by policy-makers and reflected in schemes to promote environmental management^23,24^. Policy-makers and land managers need tools which they can use to model and compare the likely effectiveness of different options before implementing conservation or other management schemes. This paper presents an example of such a method which is designed to assess the relationship between historic field boundaries and the management of soil erosion.

Several methods have been employed to measure, estimate and monitor soil erosion both at field and landscape scales^25^. Computer-based modelling can provide a quantitative and consistent approach to estimate soil erosion under a wide range of conditions. Soil erosion modelling is one of the most versatile tools for planning suitable soil protection measures and detecting erosion hotspots both at local and landscape scales^26,27^. In particular, the integration of Geographic Information Systems (GIS) with the Revisited Universal Soil Loss Equation (RUSLE) model has been widely applied to estimate soil loss in various geographical contexts^28,29^.

RUSLE is an empirical soil erosion estimation model^30^ to predict annual soil loss by sheet and rill water erosion. The RUSLE Eq. (1) needs five parameters (R—rainfall erosivity factor, MJ mm ha^-1^ h^-1^ yr^-1^; K—soil erodibility factor, Mg h MJ^-1^ mm^-1^; LS—slope length and slope gradient factor, dimensionless; C—cover management factor, dimensionless value comprised between 0 and 1; P—conservation practice factor, dimensionless value comprised between 0 and 1) for the estimation of the average annual soil loss (A) measured in tonnes/hectare/year (Mg ha^−1^ yr^−1^)^31^.

1 $$A = R*K*LS*C*P$$

In a given area the R, K and LS factors represent the environmental conditions while the C and the P factors reflect its LULC setting. Several methods were developed in recent decades to calculate the five RUSLE factors^28^. Nevertheless, the P factor remains one of the most difficult parameters to determine since detailed geospatial data about local soil conservation techniques are generally not available. Consequently, the P factor value in the RUSLE equation has sometimes been estimated by combining the land use type with the slope gradient: the lower its value, the better the effect of controlling soil erosion^32^.

Among pre-industrial agricultural practices, the creation of field boundaries (e.g. stone walls, hedgerows, earth banks, lynchets) has proved an effective means to prevent or at least limit the process of soil erosion^33^. Recently a novel P-factor model for Europe has been developed from the data retrieved during a statistical survey that recorded the occurrence of stone walls and grass margins in EU countries^34^. Although it represents one of the first attempts to consider cultural landscape features of these kinds in a soil erosion model at a continental scale, several limitations were highlighted by the authors, including the limited number of points surveyed and the interpolation technique selected^34^.

Landscape archaeology has the potential to fill this gap in the data about soil conservation practices using a GIS-based tool called Historic Landscape Characterisation (HLC)^19,35,36^. HLC is an archaeological approach which is used to map landscapes with particular reference to their long-term development by systematically interpreting their components (e.g. scale, morphology and pattern of fields, boundaries, roads, settlements, etc.)^36^. HLC permits archaeologists to record information about changing LULC by interpreting the spatial and chronological complexity of cultural landscapes.

**Figure 1:**
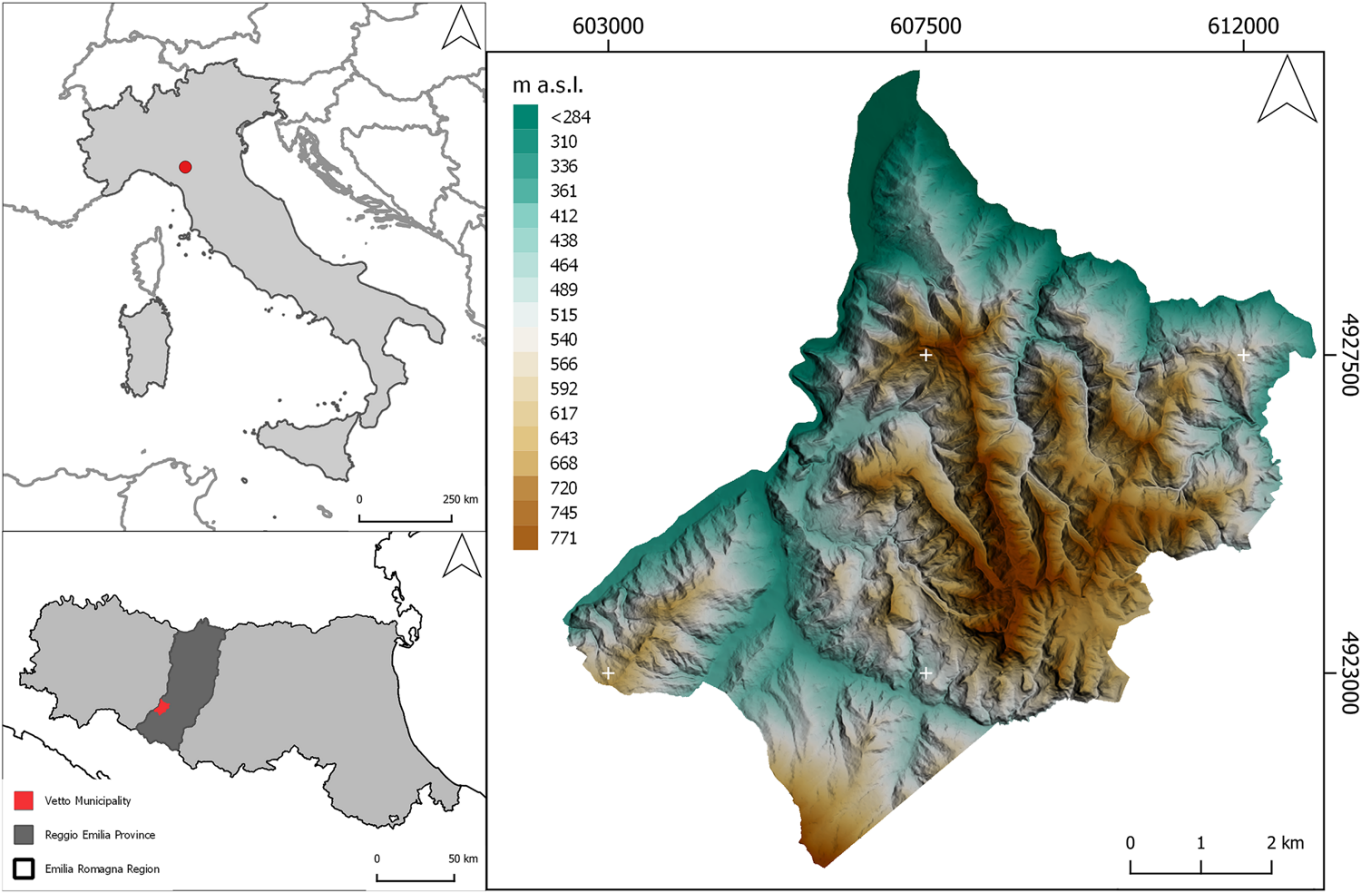
Case Study. Location of Vetto on the Northern Apennines (Reggio Emilia province, Italy). Image generated with the software QGIS 3.22 LTR (https://www.qgis.org/en/site/index.html).

The RUSLE equation has recently been employed to predict future soil erosion rate by considering the effect of climate change projections on rainfall^37^. Conversely, this research proposes an innovative method to model the effectiveness of pre-industrial agricultural features in mitigating soil erosion risk in response to the current environmental conditions. This methodology has been tested through a case study in the Tuscan-Emilian Apennines (N Italy), an area particularly susceptible to landslide hazard^38^ (Fig. 1). HLC data were integrated in the RUSLE equation using GIS software to evaluate the impact of historic rural landscape features on contemporary environmental settings. The study offers a new protocol that could be used by policymakers and stakeholders who need to develop strategies which encompass both natural and cultural values for landscape planning and management.

### Study area

The methodology was tested on a portion of the Northern Apennines (Reggio Emilia province, Italy) coinciding with the municipality of Vetto d'Enza (commonly referred as Vetto), located on the right bank of the Enza River in the Man and the Biosphere UNESCO reserve of the Tuscan—Emilian Apennines^39^ (Fig. 1). The lithological composition of the area is mainly sedimentary rocks (i.e. sandstone and marl) and geomorphological slope processes are particularly prevalent^40^. Human occupation is well-documented since the mid-Holocene^41^, but a profound reorganisation of the rural environment appears to have taken place in the Medieval period (seventh-twelfth centuries CE)^42^.

**Figure 2:**
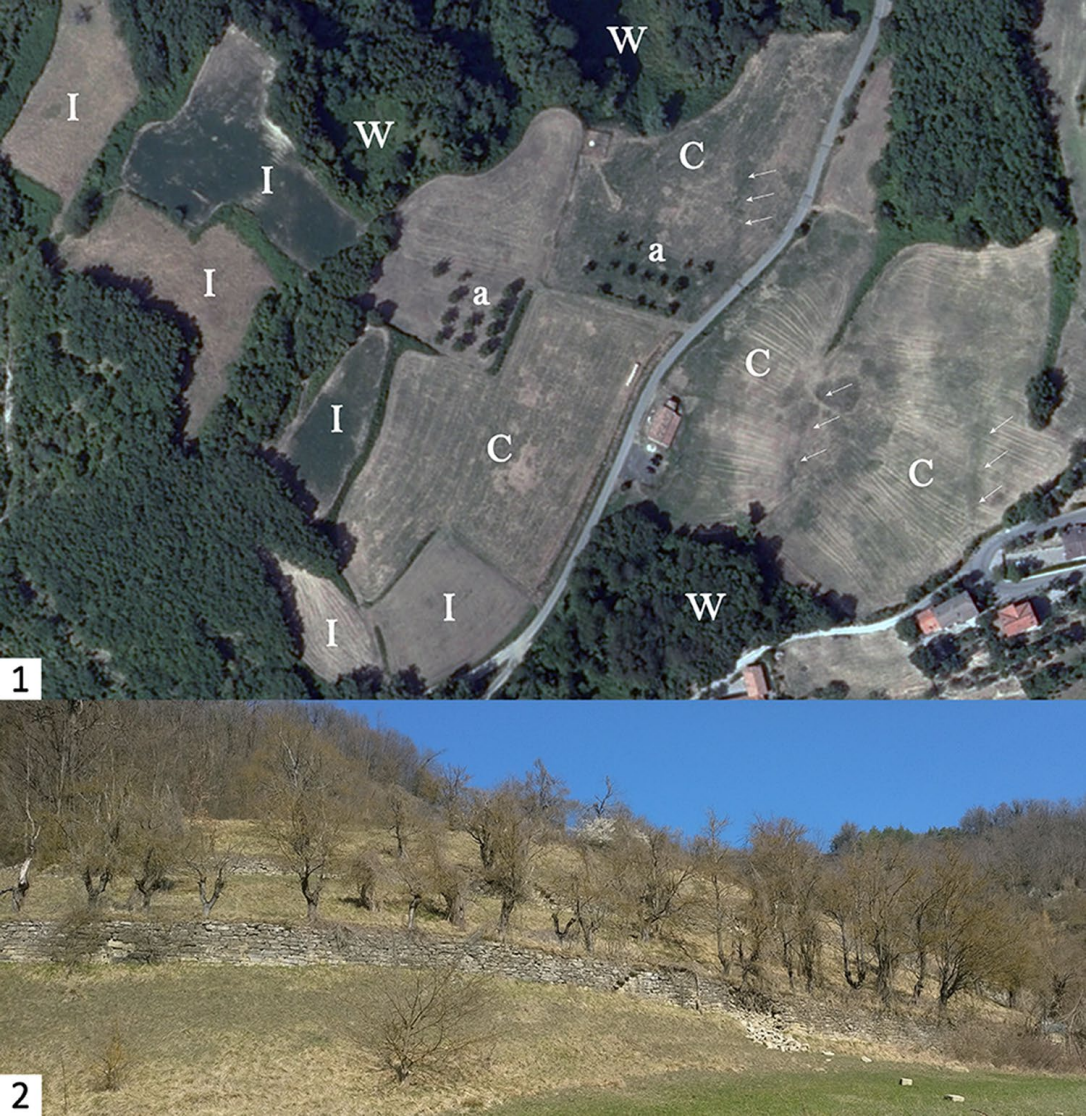
The most common historic landscape features in the study area. 1—irregular fields (I), combined fields (C), abandoned fields dominated by woodland (W), relics of agroforestry systems (a). The arrows mark past field boundaries removed to merge Irregular fields (I) into Combined fields (C). 2—historic terrace systems with abandoned agroforestry.

According to historical records, the sharecropping system was largely adopted in the region from the thirteenth century CE along with agroforestry practices (i.e. polyculture)^43^. Since the mid-twentieth century CE the decline of agrarian activities in the area has resulted in land-use change which was often related to the abandonment of farmland and a progressive depopulation of the mountains^44^. The cultural landscape of the area is still mostly characterised by a mosaic of irregular fields, which are often delimited by hedgerows with trees and shrubs (Fig. 2). Among the most distinctive characteristics of the area are well-preserved stone walls that have been used extensively between steeply-sloping fields to delimit tenurial boundaries and to face agricultural terraces (Fig. 2)^19^.

## Materials & methods

**Figure 3:**
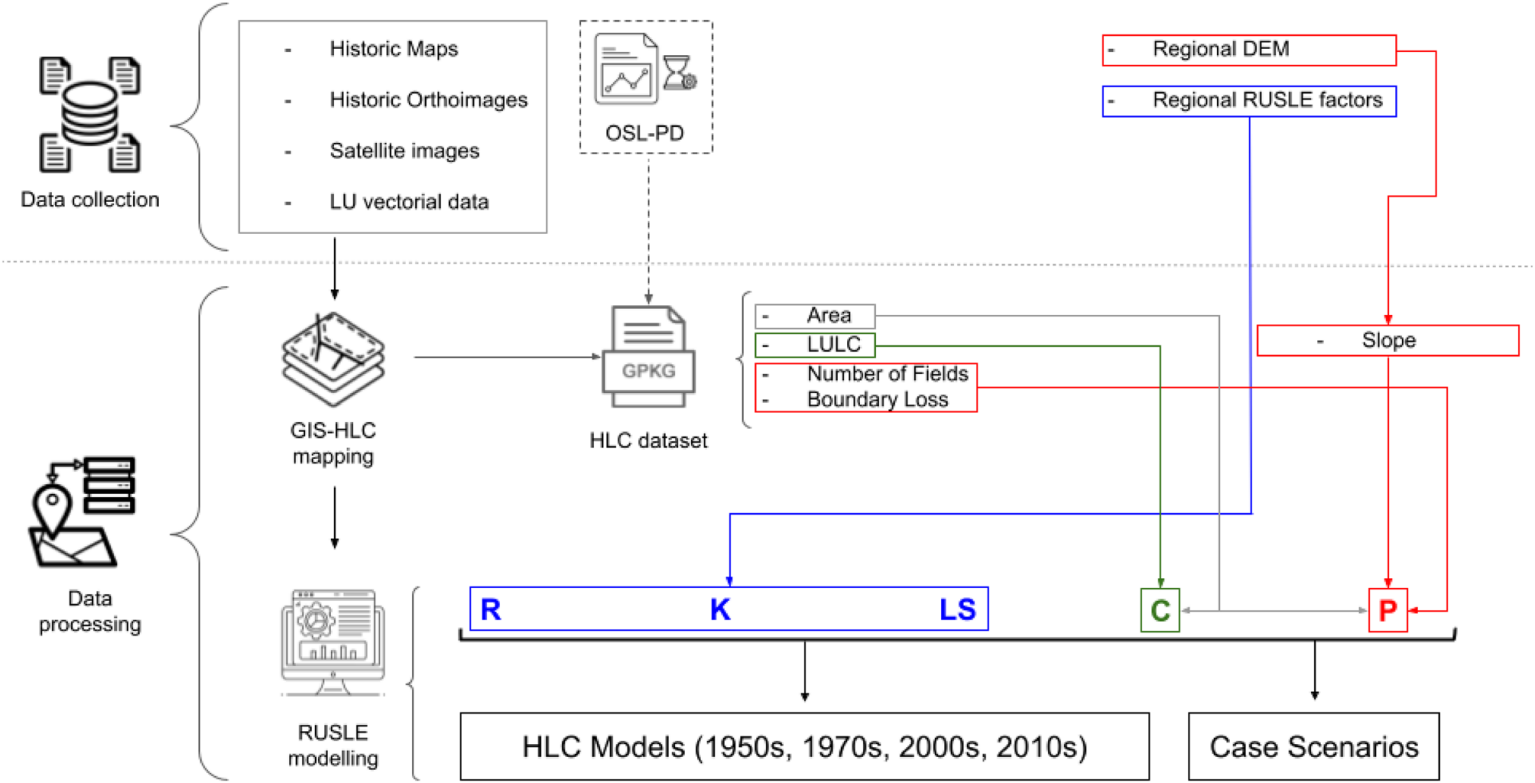
Schematic representation of the workflow employed in this research. The first step (Data Collection) was necessary to develop the HLC dataset employed in the RUSLE equation (Data Processing). Optically Stimulated Luminescence Profiling and Dating (OSL-PD) was employed to assess the chronology of historic field boundaries and agricultural terraces in the area.

The research workflow can be divided into two parts (Fig. 3). The first regards the collection and digitisation of cartographic material and remote sensing data within the open-source Geographic Information Systems (GIS) software QGIS 3.22^45^. These data sources were interpreted in GIS to create the HLC mapping of the study area^19^. Secondly, the resulting HLC dataset was employed to develop the RUSLE parameters and models within the software system for statistical computing in R^46^ through the visual interface of Rstudio^47^. For sake of clarity, in this paper the program language R is indicated as R* to avoid any confusion with the RUSLE rainfall erosivity factor R.

### Data collection: HLC dataset

Mapping an area with HLC entails the identification of ‘Uniform Diachronic Units’ (UDUs) with distinct landscape character through time (according to project-specific thresholds)^36^. Here the specific threshold chosen corresponds to a minimum area of 0.5 ha (= 5000m^2^). The UDUs were identified based on the study of various sources including historic maps, 19^th^ -century cadastral records, aerial photography and satellite images. Further data about historic land use were recovered in the regional geodatabase^48^. It is important to note that the landscape mapped in the nineteenth century regional cadastre matches largely with the 1950s fields, whose pattern likely originated in the Late Medieval period (fourteenth, fifteenth century CE)^49^. This interpretation has been validated by optically stimulated luminescence profiling and dating (OSL-PD), a methodology developed and successfully applied in several European contexts to date the sediments associated with earth banks, stone walls and agricultural terraces^22,50,51^. The results provide secure construction dates in the Middle Ages (tenth–fifteenth century CE) for most of the historic boundaries examined in the area, with evidence for further development and subdivision of the systems in the Early Modern period (fifteenth—eighteenth century CE) (see Supplementary Data 1, Table S4). The OSL profiles from the stone-walled terraces also demonstrate how effectively such features have contributed to soil conservation over extended periods (Supplementary Data 1, Fig. S1).

The resulting HLC dataset employed in this study consists of a GeoPackage (.gpkg)^52^ vectorial layer of polygons associated with an attribute table in which all the HLC characteristics are sorted. Five different periods were defined for the HLC mapping (19th -century, 1950s, 1970s, 2000s, 2010s) which covers 170 years of cultural landscape changes (Supplementary Data 2).

### Data processing: RUSLE modelling

**Figure 4:**
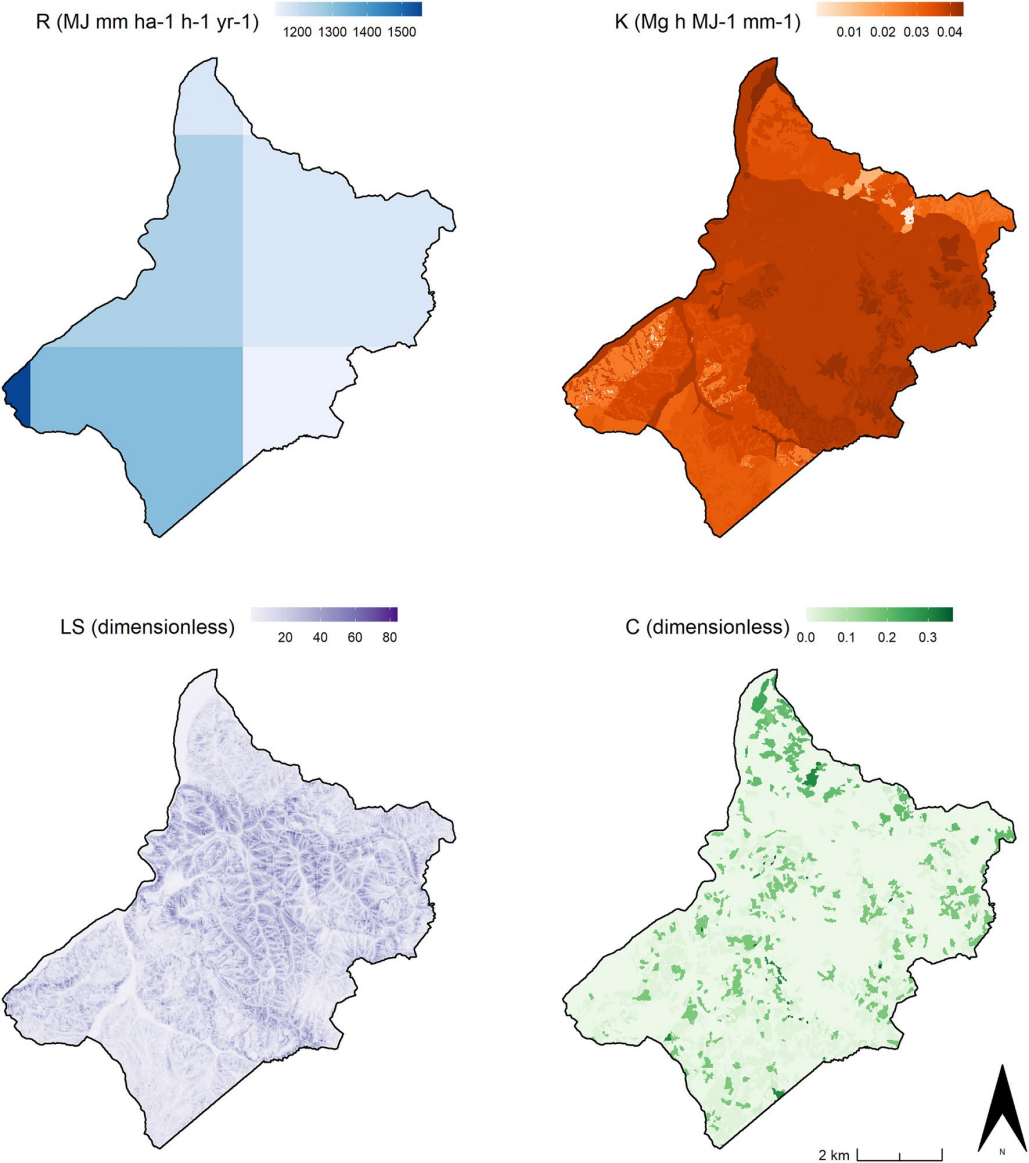
Regional RUSLE Data. From the top left to the right bottom: rainfall erosivity factor (R), soil erodibility factor (K), slope length and slope gradient factor (LS), cover management factor (C). Image generated with software RStudio Desktop 2022.07.2 + 576 (https://www.rstudio.com/).

The RUSLE environmental parameters (R, K, LS) employed in this study were supplied by the Emilia—Romagna region geological service^53^. Details about how these parameters were generated are available in the documentation provided along with the regional soil erosion map^54^. In the Emilia—Romagna 10-m resolution model, the C factor was determined using the regional 2014 land use map while local conservation strategies were not considered in the equation (i.e. P factor = 1) (Fig. 4).

In this study only the regional R, K, and LS parameters were employed while the C and the P factors were calculated in Rstudio managing the data recorded in the HLC dataset. Indeed, the main research goal is to model the effects of historic rural strategies on soil degradation over the long term, and to evaluate which historic cover management and conservation practices return the best benefit in terms of soil erosion mitigation in the contemporary environmental setting.

The cover management factor was obtained by associating the European Soil Data Centre (ESDAC) C Factor numerical values^55^ to the corresponding Regional LULC categorical data^56^ for each HLC chronological period (Table 1). The ESDAC C factor values were chosen to potentially extend the reproducibility of this protocol in other European regions.

**Table 1:** C factor values adopted in this research.

Corine LULC class^57^	C factor values^55^
5.1.2 Water bodies	0
1.1.1 Continuous urban fabric	0.0003
3.1.3 Mixed forest	0.0011
3.2.4 Transitional woodland-shrub	0.0219
3.2.1 Natural grassland	0.0435
2.4.4 Agroforestry areas	0.0881
2.3.1 Pastures	0.0903
2.4.2 Complex cultivation patterns	0.2
2.1.1 Non-irrigated arable land	0.166^58^
2.2.2 Fruit trees	0.2188
2.2.3 Olive graves	0.2273
3.3.3 Sparsely vegetated areas	0.2652
2.2.1 Vineyards	0.3527

The calculation of the P factor using landscape archaeological data required a more sophisticated map algebra. Starting from the assumptions that (i) the construction of field boundaries has always represented an effective method to limit soil erosion^33^ and that (ii) the efficiency of any conservation measures to mitigate soil erosion increases with the increasing of the slope^59^, this P factor Eq. (2) has been developed:

2 $$P=\frac{(\frac{A}{N}*{A}^{-1})}{tan {S}_{mean}}$$

where A is the HLC polygon area (m^2^), N is the number of fields in A, and S is the mean slope gradient (in %) of the HLC polygon. The numerator consists of the ‘field concentration’ of the HLC polygon and its value is comprised between 0 (i.e. ∞ fields / polygon) and 1 (i.e. 1 field / polygon). Conceptually, in a given rural area, the higher the number of fields (i.e. the number of field boundaries), the lower the P Factor value should be (i.e. soil erosion mitigation is greater). On the other hand, the denominator represents the gradient of inclination expressed as the tangent to the mean slope of each HLC polygon.

**Figure 5:**
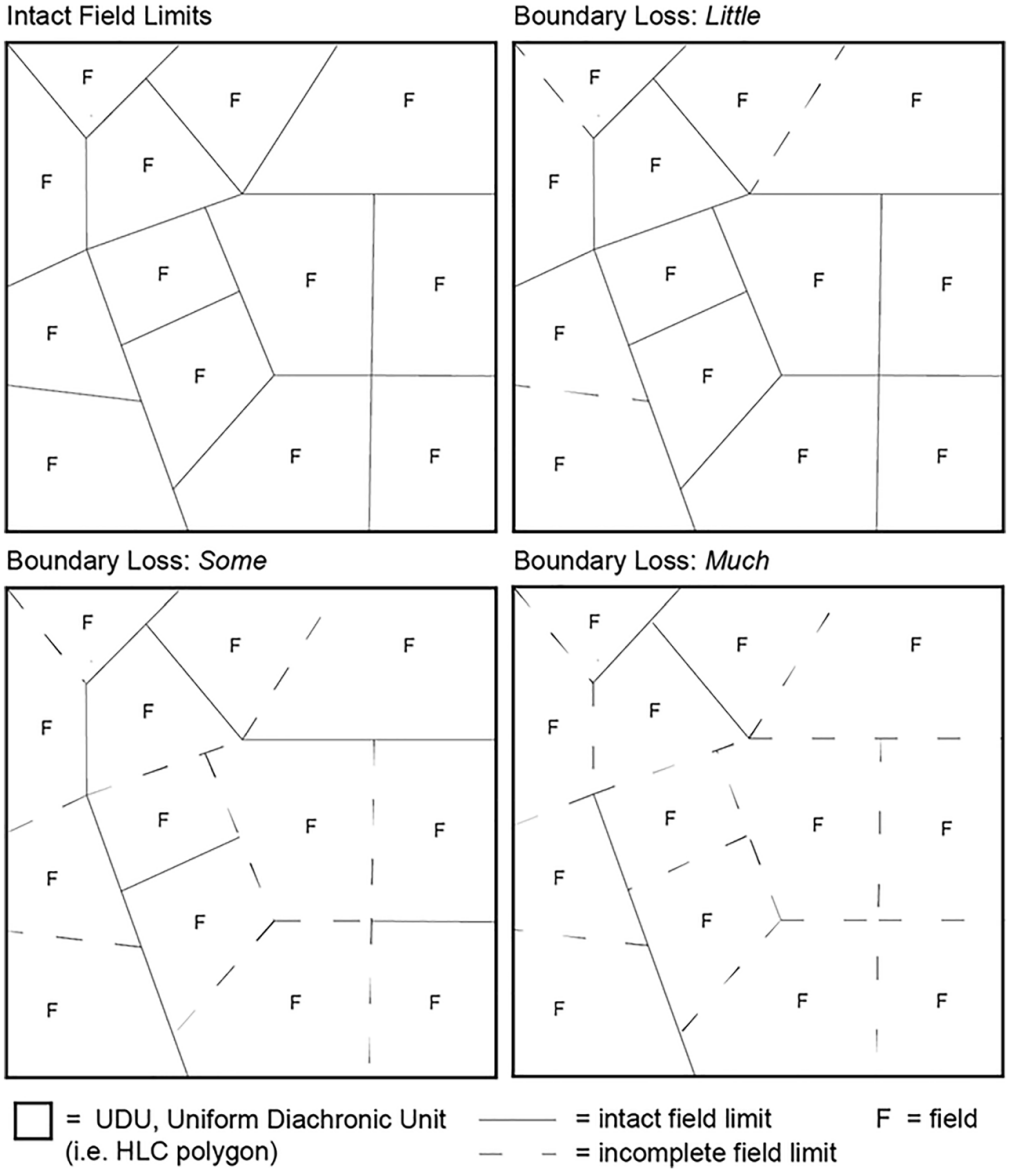
Schematic representation of the *‘Boundary Loss’* attribute used in the Historic Landscape Characterisation analysis.

The regional 5-m resolution Digital Elevation model^48^ was analysed with the r.slope.aspect GRASS^60^ module in QGIS to achieve the Slope values. Then the *S*_*mean*_ values for each HLC polygon were extracted with the QGIS Zonal Statistics plugin. During the development of the HLC dataset a different degree of conservation was observed in field limits so the P factor has been ‘*calibrated*’ using the HLC attribute ‘*Boundary Loss*’. The reasoning behind this assumption is that the P factor should increase as the ‘*Boundary Loss*’ is more evident. In other words, given two vectorial polygons with the same number of fields, the polygon in which soil loss retention is expected to be higher corresponds to the one in which the field limits are better preserved. In the HLC dataset three degrees of *‘Boundary Loss*’ have been considered*: Little*, *Some* and *Much* (Fig. 5).

Intact field boundaries are likely to be much more effective in retaining soil erosion than poorly preserved limits, such as vegetative barriers^61^. This is demonstrated in our case study at Vetto, were OSL-PD was used to fully characterise the sediment stratigraphies preserved behind the terrace walls and in the earth banks. This technique allows one to develop relative chronologies and compare / contrast sediments in time-depth. The terrace stone walls have been effective in retaining soils over a dynamic range of 9 to 11, implying that these soils have been aggrading over a considerable time (see Supplementary Data 1).

Thus, to include the ‘*Boundary Loss*’ in the P factor modelling, corresponding increasing percentages for each ‘*Boundary Loss*’ category were proposed (Table 2).

**Table 2:** Boundary loss values proposed in this research.

Boundary loss	P factor increasing (%)
Much	+ 80
Some	+ 40
Little	+ 20

In the study area the only support practice sub-factor registered is terrace farming so its efficiency in mitigating soil erosion was calculated in the P factor according with the method proposed by *Parveen and Kumar 2012*^62^.

The C and P parameters were developed using the R* package for data manipulation dplyr^63^ and then converted in rasters file within stars^64^, an R* package to manage spatiotemporal arrays. Finally, the RUSLE models for four chronological phases (1950s, 1970s, 2000s, 2010s) were calculated combining the regional rainfall erosivity (R), soil erodibility (K), and topographic (LS) factors with the HLC C and P parameters using Eq. (1). The resulting RUSLE models consist of Geostationary Earth Orbit Tagged Image (GeoTIFF) files with a grid resolution of 10 m. In the absence of reliable LULC data to develop the C factor for the 19th -century chronological phase, this period has not been considered in the soil erosion modelling. Still, the P factor generated using the 19^th^ -century HLC data was employed to simulate the effect of pre-industrial fields' pattern in possible case-limit scenarios (see Discussion). Furthermore, the R* package for statistical calculations stats^47^ was employed to predict future P factor values in the 2050 to simulate the interaction of cultural landscape features with the latest projections on climate change (see Discussion).The Results and Discussions sections are supported with thematic maps and illustrations generated in Rstudio with the package ggplot2^65^. In order to enable reproducibility, the HLC dataset and the R* code used for this project are provided as supplementary material in R* Markdown format (see Data availability statement).

## Results

**Figure 6:**
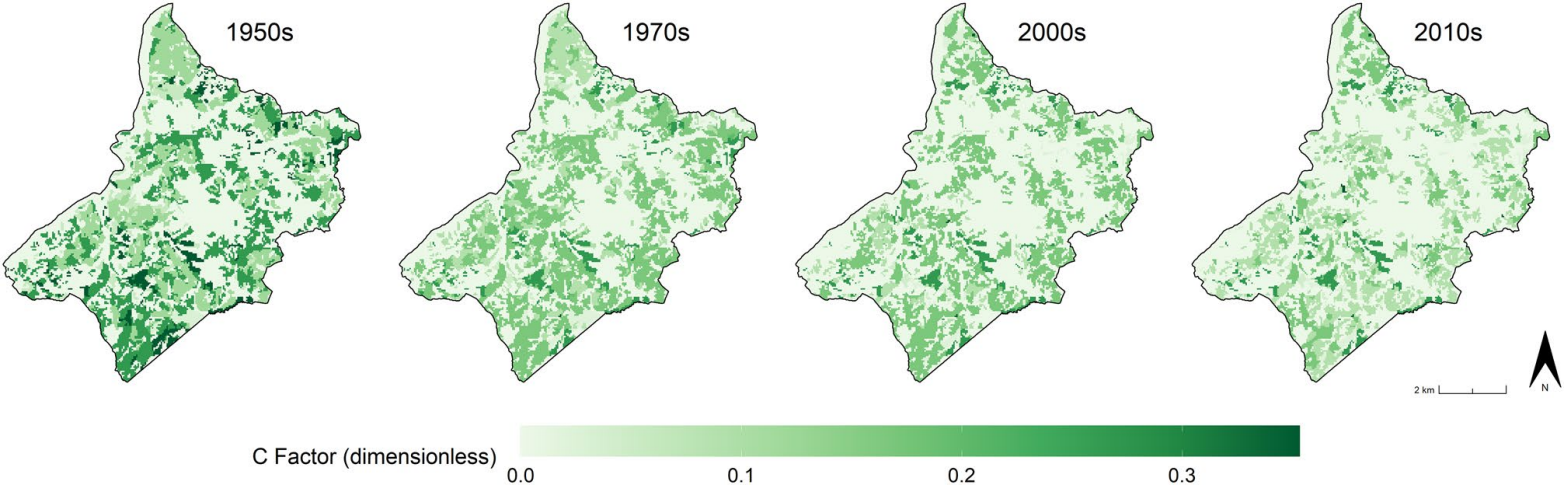
The cover management (C) factors calculated for the four chronological periods: 1950s, 1970s, 2000s, 2010s). Image generated with software RStudio Desktop 2022.07.2 + 576 (https://www.rstudio.com/).

The integration of the HLC dataset within the RUSLE equation returns remarkable results about the effects of cultural landscape changes on soil erosion risk in the area over the last 70 years. Firstly, the cover management (C) factors calculated for the four chronological periods show a constant decrease since the 1950s (Fig. 6).

**Figure 7:**
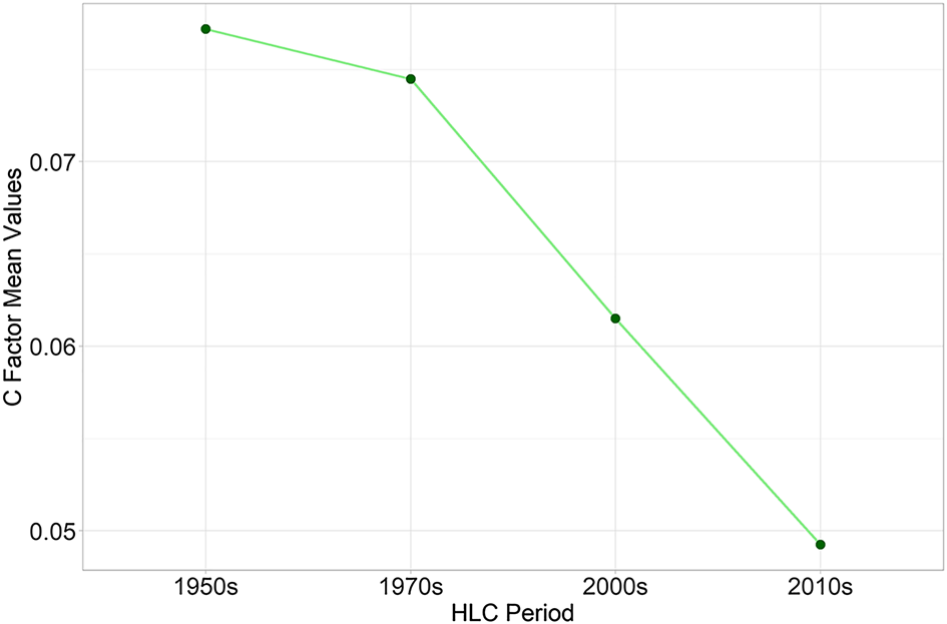
The C factors mean values in the four chronological periods: 1950s, 1970s, 2000s, 2010s). Image generated with software RStudio Desktop 2022.07.2 + 576 (https://www.rstudio.com/).

Looking at the mean values of each period a clear lowering trend in the C factor values especially after the 1970s is observable (Fig. 6). This reflects two particular socio-economic trends in the area since the mid-twentieth century CE: the progressive reduction of rural activities in mountainous regions^66^ and the need for forage for the regional dairy industry^67^. As a consequence, the historic polyculture systems and the mosaic of small cultivated land parcels were replaced by woodland and pastures that present lower C factor values than other agriculture practices (Fig. 7). Thus, the cultural landscape changes return an overall reduction of 37% of the mean C factor values in the study area. In other words, considering the contemporary environmental conditions (i.e. the regional R, K, LS factors), the current cover management setting in the area (i.e. HLC 2010s) seems to be less severe in terms of soil erosion hazard than the historic rural LULC registered in the HLC 1950s.

**Figure 8:**
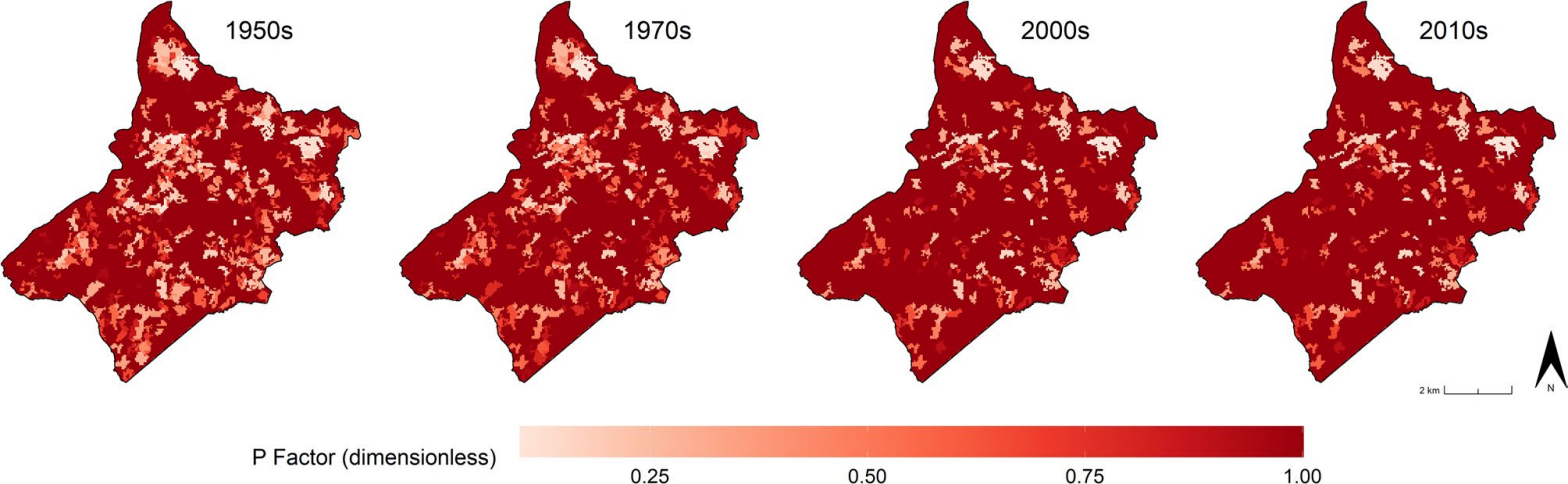
The conservation practice (P) factors calculated for the four chronological periods: 1950s, 1970s, 2000s, 2010s). Image generated with software RStudio Desktop 2022.07.2 + 576 (https://www.rstudio.com/).

**Figure 9:**
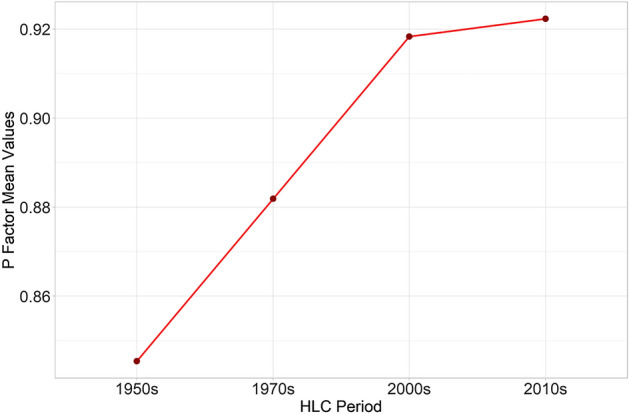
The P factors mean values in the four chronological periods: 1950s, 1970s, 2000s, 2010s). Image generated with software RStudio Desktop 2022.07.2 + 576 (https://www.rstudio.com/).

Secondly, as done for the C factor, the conservation practice parameter has been calculated for the four HLC periods (Fig. 8), and the corresponding P factor mean values reveal a rising trend from the 1950s to the 2010s (Fig. 9). This is largely due to the replacement of patches of pre-industrial irregular fields with larger open fields created by removing physical field boundaries. Even when traditional field margins are still recognisable, they are often in a poor state of conservation. In general, the P factor in the study area increased by 9.7% since the 1950s.

**Figure 10:**
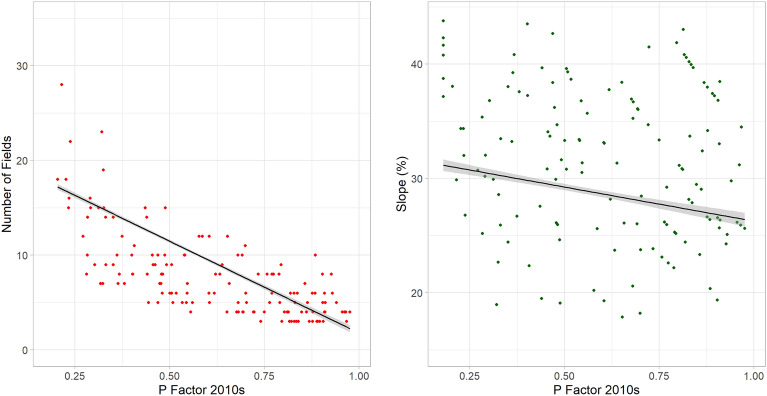
The 2010s’ P factor values compared with 2010s’ Number of Fields and Slope (%) trends. Image generated with software RStudio Desktop 2022.07.2 + 576 (https://www.rstudio.com/).

To test the goodness-of-fit of the P factor equation proposed in this study (2), the conservation practice values were compared with the corresponding number of fields and the slope gradient. As expected, the P factors values present an inverse correlation with them: when the boundaries and the inclination gradient lowers, the conservation practice is less effective (i.e. P increases) (Fig. 10).

The C and P parameters generated were employed to estimate the soil erosion hazard in the four historic periods (i.e. HLC 1950s, 1970s, 2000s, and 2010s) according to the current environmental settings (regional R, K, LS factors).

**Figure 11:**
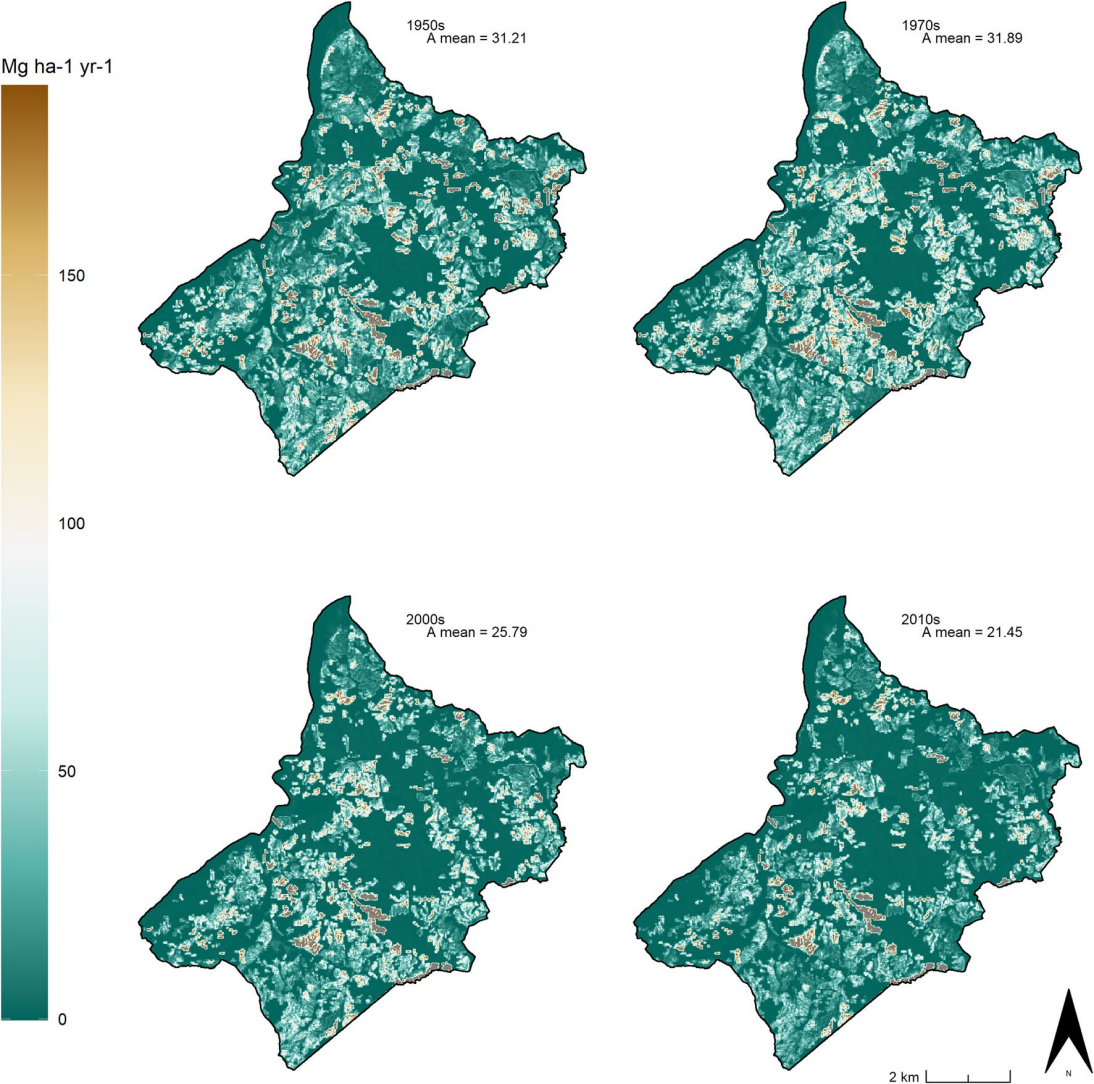
The HLC-RUSLE model calculated for the four chronological periods: 1950s, 1970s, 2000s, 2010s). Image generated with software RStudio Desktop 2022.07.2 + 576 (https://www.rstudio.com/).

**Figure 12:**
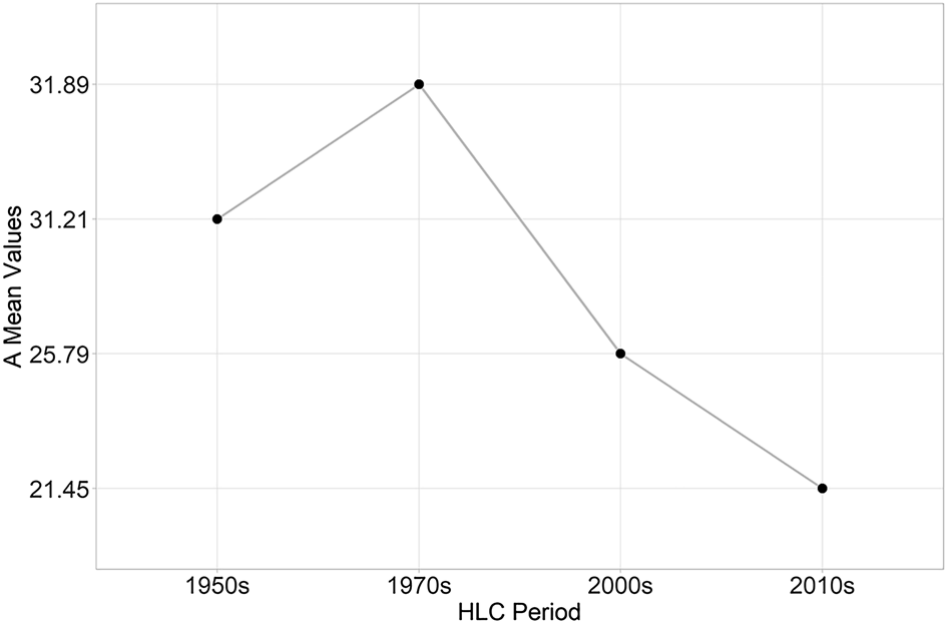
The A mean values in the four chronological periods: 1950s, 1970s, 2000s, 2010s). Image generated with software RStudio Desktop 2022.07.2 + 576 (https://www.rstudio.com/).

The resulting RUSLE models (10-m resolution) show a general decrease of the soil loss estimation in the study area over the last 70 years (Fig. 11). The highest A mean value (Fig. 12) corresponds to the HLC 1970s in which the cover management factors were almost the same as was in the previous period (Fig. 7) but the conservation practices were already less effective than in the HLC 1950s (Fig. 9).

## Discussion and conclusions

**Figure 13:**
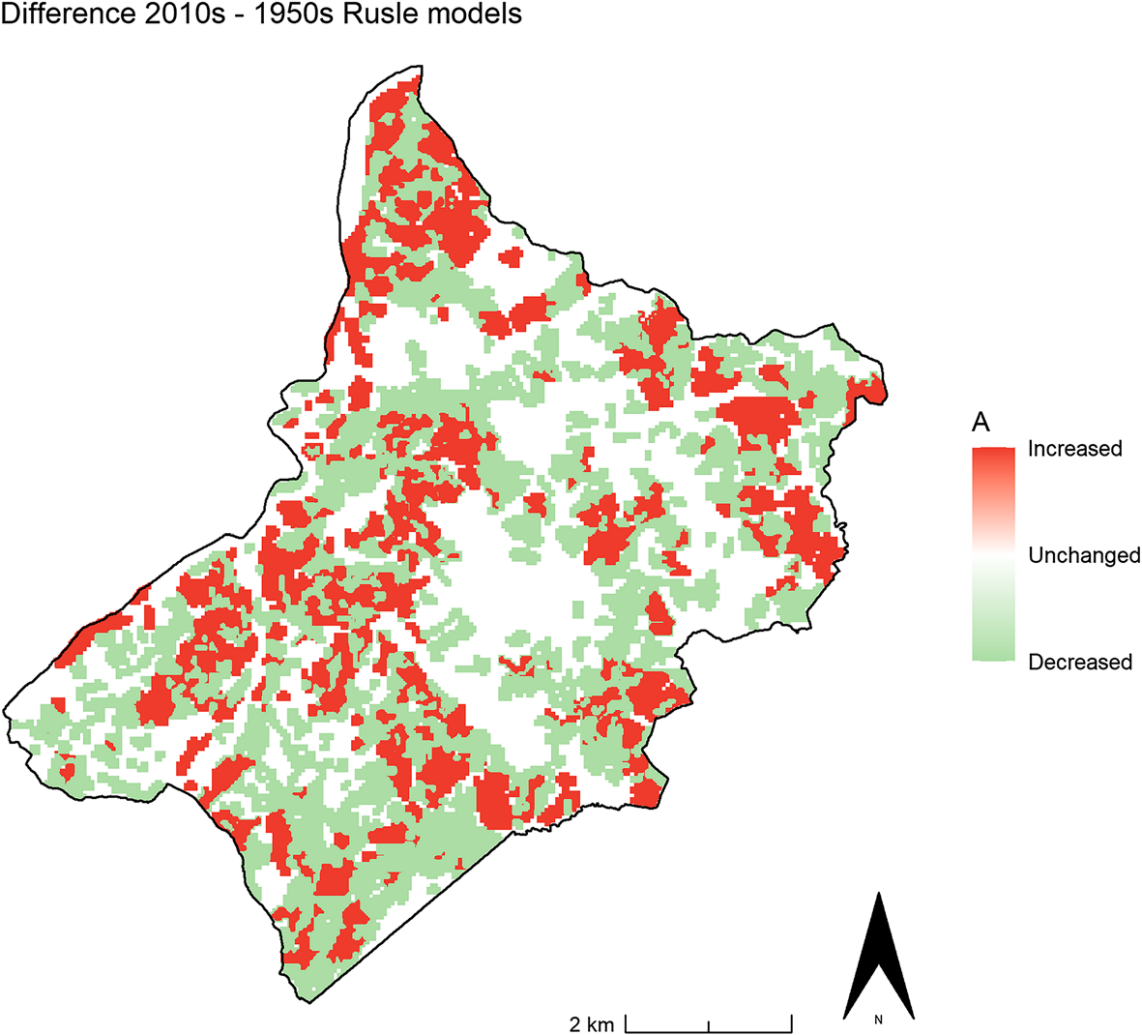
The comparison between the 2010s and the 1950s HLC-RUSLE models highlights zones where A increased/decreased or remained unaltered). Image generated with software RStudio Desktop 2022.07.2 + 576 (https://www.rstudio.com/).

According to the Results, it seems that the pre-industrial rural know-how was less effective in mitigating soil erosion than modern agriculture, an assumption that is exactly the contrary of what is outlined in scientific literature^20,33,34^ as well as in inter-governmental organisation guidelines^3,68,69^. In fact, the general trend shows a significant decrease in the overall value of A in the study area as the historic LULC characteristics changed in the last 70 years (Fig. 12), but this general reduction is strongly due to the progressive rewilding and depopulation processes that have been affecting the mountainous regions since 1950s^70^. Indeed, with a map algebra subtraction of the HLC 2010s RUSLE model with the HLC 1950s RUSLE model, the situation appears much more complex and heterogeneous (Fig. 13), highlighting the effects of the historic landscape changes on the soil erosion risk of the area. Where A is unchanged corresponds to areas in which the C and the P factors remained unaltered throughout the past seven decades: these areas are largely forests. On the other hand, the green zones (i.e. where A decreased) pertain to parts of the landscape in which historic rural activities were replaced with an agriculture type that has a lower C factor (e.g. arable land replaced by pastures), were abandoned (e.g. woodland) or were affected by urbanisation processes (e.g. residential areas or industrial zones) (Table 2). Finally, red zones (i.e. where A increased) widely belong to modern open fields created by removing traditional margins (e.g. hedgerows, earthbanks, lynchets, etc.) and where the pre-industrial polyculture strategies (i.e. agroforestry) were replaced with monoculture arable land or pastures (Fig. 13).

**Figure 14:**
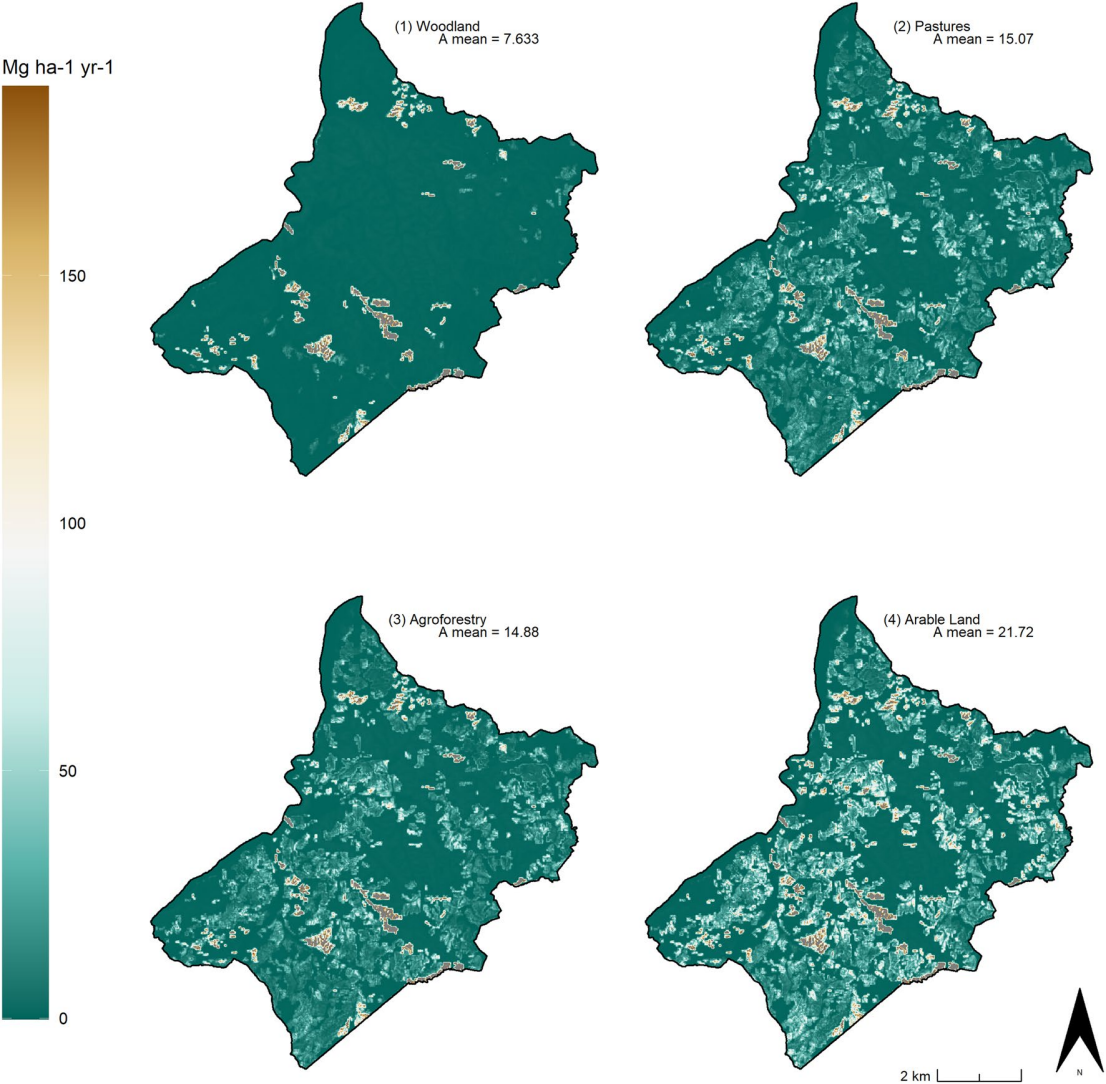
Case-limit scenarios: 1- Woodland, 2- Pastures, 3- Agroforestry, 4- Arable Land). Image generated with software RStudio Desktop 2022.07.2 + 576 (https://www.rstudio.com/).

Starting from the observations highlighted in Fig. 13, four possible case-limit scenarios were simulated (Fig. 14). Three considered the C factor for each of the three main historic agricultural LULC types recorded in the area (i.e. pastures, agroforestry, arable land) and the pre-industrial P Factor developed from the 19th-century cadastral maps. These three models were compared with a further scenario (Scenario 1) that simulates the soil erosion risk if all the current rural activities were abandoned (i.e. P factor = 1) and replaced only by woodland (i.e. C factor = 0.0011). The second (Scenario 2) estimated the soil erosion risk in the case in which all the current farmland was only dedicated to forage production (pastures) and the historic conservation practices were intact. The third case-limit (Scenario 3) represented a scenario in which the present-day rural landscape was occupied only by pre-industrial polyculture activities and the 19^th^ -century field margins were perfectly preserved. In the last scenario (4) the pre-industrial conservation measures were still considered as if they remained unaltered but ‘arable land’ was the only rural LULC type in the area.

Scenario 1 scored the lowest A mean values representing a kind of pristine state of the study area in which the soil erosion is extremely low and concentrated only in correspondence to badlands. Conversely, the theoretical scenarios 2, 3 and 4 are particularly helpful in highlighting the environmental impact of each LULC category in combination with the historic field pattern registered in the nineteenth century cadastral maps. Scenario 2 and 3 return relatively low A values, while the A value in Scenario 4 is much higher (Table 3). In Scenario 4 ploughing would increase the magnitude of runoff on slope farmlands resulting in land degradation^71^. Meanwhile the complete replacement of agroforestry systems with pastures (Scenario 2) or monoculture arable land (Scenario 4) would both represent a significant loss in the historic character of the study area.

**Table 3:** A, C and P factors’ mean values of the HLC—RUSLE models compared with the four simulated case-limit scenarios. The values of Scenario 3 are in [bold].

Case	C Factor (Mean)	P Factor (Mean)	A (Mean)
HLC 1950s	0.07718	0.8454	31.21
HLC 1970s	0.07447	0.8819	31.89
HLC 2000s	0.06149	0.9183	25.79
HLC 2010s	0.04925	0.9223	21.45
Scenario (1)	0.01118	N.A	7.633
Scenario (2)	0.03974	0.8391	15.07
**Scenario (3)**	**0.03904**	**0.8391**	**14.88**
Scenario (4)	0.06453	0.8391	21.72

The latest agricultural European Innovation Partnership (EIP-AGRI) workshop considers reconciling production with the sustainable management of land as being the major challenge for current and future agriculture systems^69^. In addition, the recent pandemic and conflict in Europe have highlighted the desirability of less globalised food production^72,73^. Polyculture systems have existed since ancient times in Europe (e.g. *dehesa* in Spain; *montado* in Portugal and *streuobst* in Germany) and European policies encourage the preservation and creation of agroforestry systems due to their 'high ecological and socio-cultural value'^74^. Indeed, agroforestry has been widely recognised^68^ as a sustainable land-use system that addresses food production needs while providing various environmental benefits to society (e.g. reduction of CO_2_ emissions, increase of carbon sequestration, soil erosion mitigation)^75^. Thus, with a relatively low impact on soil erosion, Scenario 3 seems to represent a good compromise between food production, climate change mitigation and the restoration of historic landscape character.

Despite all these potential environmental and archaeological advantages, Scenario 3 remains a simulation and its realistic application would need to take into account other variables such as the short-term economic feasibility of reconverting modern agriculture to a pre-industrial status, the possible increases in wild-fire risk due to the large-scale replacement of arable land and pastures with agroforestry and the short-term ecological impact on local fauna. Environmentally sustainable rural LU types very often do not provide an immediate and desirable economic return to farmers and these solutions need to be implemented and adapted to meet site-specific needs at the local scale by inviting stakeholders to contribute to policy development^76^. Nevertheless, EU-funded research focused on polyculture and intercropping strategies have shown a return-on-investment time of about five years to recover from workforce training and machinery costs^77^. The complete conversion of an economic system requires time and constant financial support, but it has been demonstrated that even the partial integration of historical agroforestry systems within arable lands and pastures can contribute strongly to lowering environmental pressures on farmland^78^.

**Figure 15:**
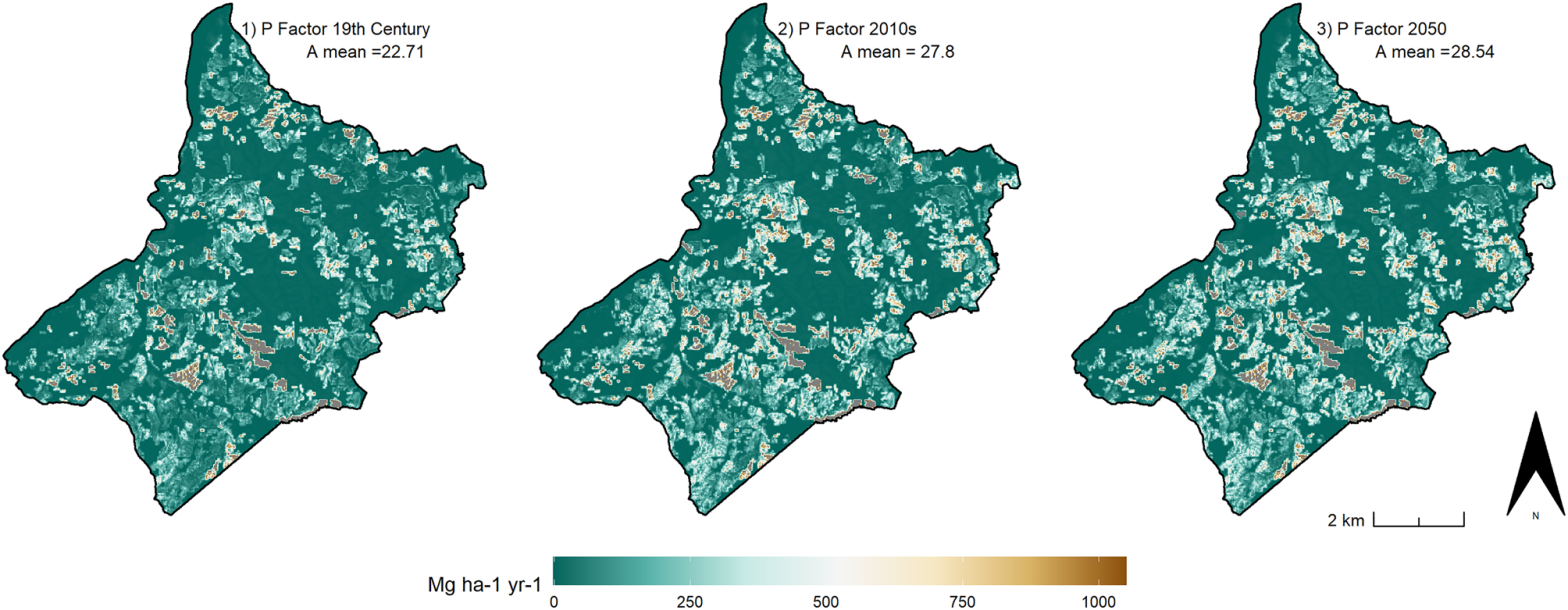
The impact that different degrees of conservation of historic landscape features would have on soil erosion based on latest projections on global rainfall in 2050). Image generated with software RStudio Desktop 2022.07.2 + 576 (https://www.rstudio.com/).

Hypothetical scenarios 2, 3, and 4 highlight how effective the historic field boundaries would have been at reducing the soil erosion hazard if they had been maintained as they were in the 19th -century (Table 3). Even in the worst Scenario in terms of possible land degradation (Scenario 4), the restoration of the pre-industrial historic field pattern would lower the soil erosion risk to a level that is only slightly higher than current values (HLC 2010s) (Table 3). To test the possible benefits that historic landscape features might provide in a future scenario, three further RUSLE models were developed considering the global rainfall erosivity projections for the year 2050^79^. The three models (Fig. 15) simulate the effect of the 2050 rainfall projection (i.e. R Factor 2050) on the current LULC systems (C Factor 2010s) if: 1) the 19^th^-century boundary patterns remained intact (i.e. P factor 19th-century); 2) the current field boundaries and agricultural terraces were unaltered (i.e. P factor 2010s); 3) the degree of deterioration of boundary features were to continue in the future as it has been over the last 70 years (i.e. P Factor 2050, developed by fitting a linear regression model with the HLC P factors). These simulations outline the potential importance of historic boundary patterns in controlling soil loss by water erosion in relation to the future increase of storm intensity and runoff (Fig. 15).

To conclude, modelling landscape archaeological data in a soil loss estimation equation enables deeper reflection on how historic strategies for soil management might relate to current environmental and climate conditions. International treaties and policies^80,81^ outline the importance of managing landscape change in response to social needs, economic activities and environmental processes. Meanwhile, in the last ten years, the EU has encouraged re-population in rural mountain areas by providing economic incentives to newcomers who choose to relocate from cities^74^. The aim of such a policy is to limit the process of de-population, thereby avoiding the loss of cultural identity in rural regions and lowering population pressure in urban areas^82^. In response to such policies, modelling tools like the one outlined in this paper could be used to inform the design of management plans which mitigate land degradation whilst preserving the landscape character and cultural identity of an area. In this rapidly changing world, landscape archaeology has the potential to participate actively in the debate around global emergencies thanks to approaches that facilitate the dialogue with other disciplines and make knowledge of the past useful for the future.

## Data availability

HLC Data and R script code are available in Supplementary Information 2 and alternatively on Zenodo: 10.5281/zenodo.6622607.

## Supplementary Information

Supplementary Information 1.

Supplementary Information 2.
