# Supplementary material for: Modelling the impact of historic landscape change on soil erosion and degradation
Source: Sci Rep. 2023 Mar 27;13:4949. doi: 10.1038/s41598-023-31334-z (PMC10042871; doi:10.1038/s41598-023-31334-z)
Supplement: Supplementary file 1 — Supplementary Information 1. [file 41598_2023_31334_MOESM1_ESM.docx]

Supplementary data

## 1. OSL PD Analysis and Results

These supplementary datafiles concern the optically stimulated luminescence (OSL) investigations undertaken to date the constructional sequence(s) to the system of terraces and earthworks located in Vetto.

The methodological approach of Turner et al. (2021) and Kinnaird et al. (2017) was implemented. In the *first stage*, portable OSL equipment was used to appraise the luminescence properties of bulk sediment in the field (Sanderson and Murphy, 2010). This data was used to calculate OSL and IRSL net signal intensities, OSL and IRSL depletion indices and the IRSL:OSL ratio for each sample. This proxy data was plotted vs depth in the stratigraphy, and used to aid in the interpretation of the depositional sequences and position samples for quantitative quartz OSL dating. In the *second* stage, a sub-set of these samples were progressed to laboratory analysis, to provide the first approximations of apparent dose, both in terms of magnitude and range. In the *third* stage, those samples identified as having significance for reconstructing the site history are progressed to full quantitative quartz OSL dating. To collect OSL dating samples, stainless steel tubes (~4 cm diameter, ~15 cm length) were hammered into cleaned sediment faces and immediately stored in opaque bags. Bulk sediment from within 30 cm the dating position was collected for additional dosimetry measurements.

Further details on the protocols / procedures used in each of these steps are provided below.

***Stage 1: characterising the luminescence of bulk sediment in the field***

Figure S.1 shows the luminescence stratigraphies that were generated for the sediments associated with the terrace walls and earthworks at Vetto. These plots show the variation in OSL net signal intensities with depth. Table S.1 lists IRSL and OSL signal intensities, IRSL and OSL depletion indices and the IRSL: OSL ratio for each bulk sediment sample characterised with the portable OSL equipment.

In well-bleached sediments, OSL net signal intensities might act as a proxy for age (Munyikwa et al., 2021; Turner et al., 2021): higher signal intensities indicate older sediment, lower signal intensities younger sediment. One might expect signal intensities to increase with depth i.e. a normal signal-depth progression; deviations down-profile trend indicate the parts of the profile that contain sediments that were re-deposited without the luminescence being reset.

These profiles illustrate how the system of terracing the slope, by the construction of stone-built terrace walls and earth banks, have aided in the retention of soil and prevented soil erosion. In terms of time-depth, the dynamic range in OSL signal intensities between the young and old soils is in the region of x 9-11, suggesting that these soils preserve a substantial chronology.

**Figure S.1** *-* terracing on the slopes north of Vetto retain soil and prevent erosion. The five profiles show the variation in luminescence with depth for sediments associated with three terrace walls and two earth banks.


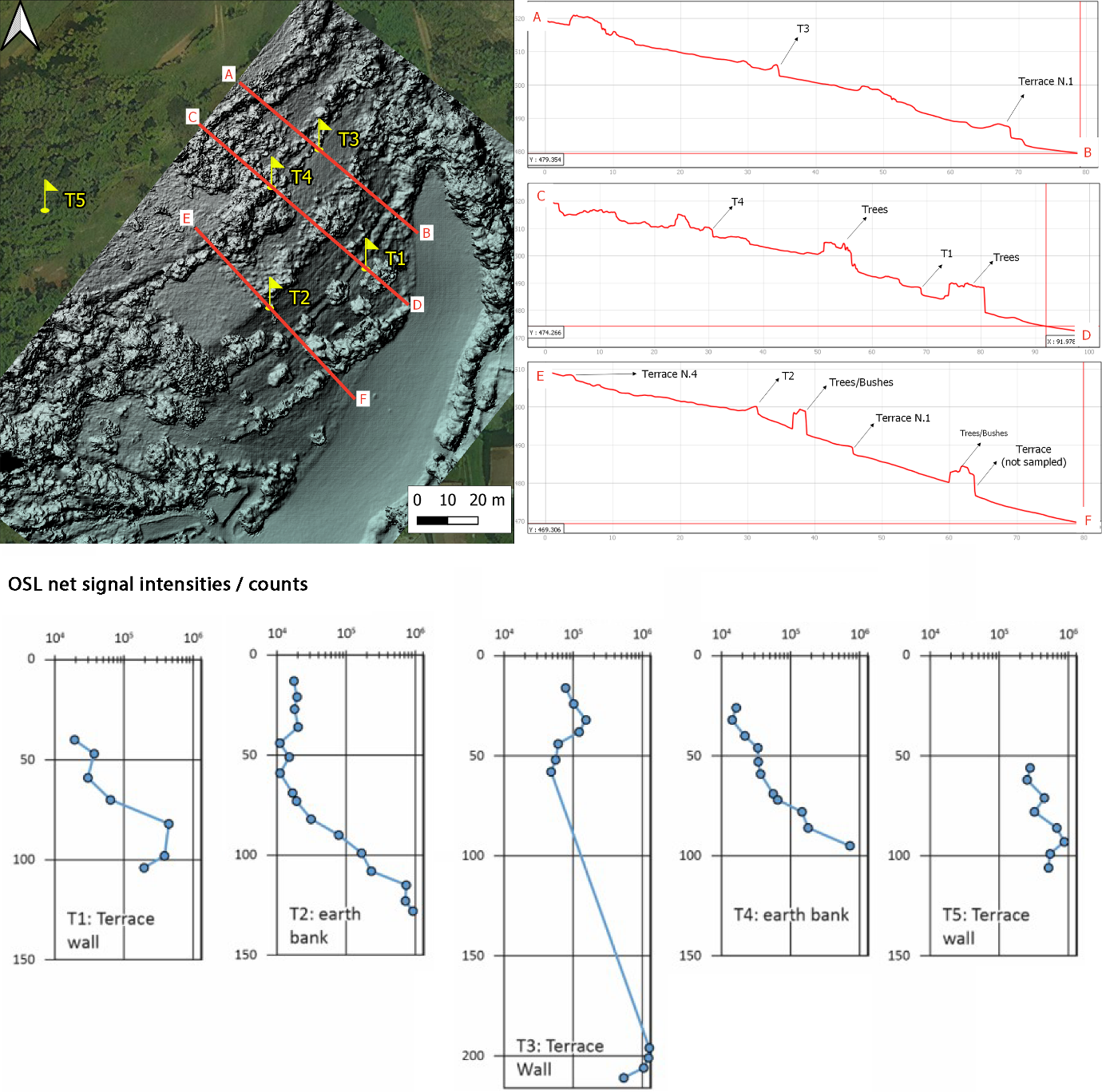


**Table S.1** *- Luminescence proxy data obtained using a SUERC portable OSL reader. The rows are colour coded by intensity: warmer colours represent higher intensities and the older sediment; cooler colours, lower intensities and the younger sediment.*

| Field ID | Depth / cm | IRSL net signal intensities / counts | IRSL depletion | OSL net signal intensities / counts | OSL depletion | IRSL : OSL ratio |
| --- | --- | --- | --- | --- | --- | --- |
|  | | | | | | |
| vs-1-1 | 40 | 28010 ± 170 | 1.31 ± 0.02 | 191850 ± 440 | 1.48 ± 0.01 | 0.1460 ± 0.0009 |
| vs-1-2 | 47 | 48930 ± 230 | 1.35 ± 0.01 | 370230 ± 610 | 1.53 ± 0.01 | 0.1321 ± 0.0006 |
| vs-1-3 | 59 | 53360 ± 230 | 1.39 ± 0.01 | 300260 ± 550 | 1.57 ± 0.01 | 0.1777 ± 0.0008 |
| vs-1-4 | 70 | 109400 ± 330 | 1.39 ± 0.01 | 638120 ± 800 | 1.62 ± 0.01 | 0.1714 ± 0.0006 |
| vs-1-5 | 82 | 969810 ± 990 | 1.42 ± 0.01 | 4456550 ± 2120 | 1.67 ± 0.01 | 0.2176 ± 0.0002 |
| vs-1-6 | 98 | 856650 ± 930 | 1.39 ± 0.01 | 3861730 ± 1970 | 1.63 ± 0.01 | 0.2218 ± 0.0003 |
| vs-1-7 | 104 | 367490 ± 610 | 1.40 ± 0.01 | 1948330 ± 1400 | 1.73 ± 0.01 | 0.1886 ± 0.0003 |
| vs-1-8 | 19 | 66920 ± 260 | 1.45 ± 0.01 | 390600 ± 630 | 1.69 ± 0.01 | 0.1713 ± 0.0007 |
| vs-1-9 | 29 | 174670 ± 420 | 1.41 ± 0.01 | 850640 ± 930 | 1.65 ± 0.01 | 0.2053 ± 0.0005 |
| vs-1-10 | 42 | 319320 ± 570 | 1.33 ± 0.01 | 1767220 ± 1330 | 1.65 ± 0.01 | 0.1807 ± 0.0003 |
| vs-1-11 | 31 | 71910 ± 270 | 1.42 ± 0.01 | 427310 ± 660 | 1.59 ± 0.01 | 0.1683 ± 0.0007 |
| vs-1-12 | 38 | 63120 ± 250 | 1.43 ± 0.01 | 361350 ± 600 | 1.66 ± 0.01 | 0.1747 ± 0.0008 |
| vs-1-13 | 49 | 112230 ± 340 | 1.43 ± 0.01 | 562750 ± 750 | 1.65 ± 0.01 | 0.1994 ± 0.0007 |
| vs-1-14 | 61 | 868020 ± 940 | 1.42 ± 0.01 | 4274960 ± 2070 | 1.73 ± 0.01 | 0.2003 ± 0.0002 |
|  | | | | | | |
| vs-2-1 | 13 | 30720 ± 180 | 1.36 ± 0.02 | 176120 ± 420 | 1.47 ± 0.01 | 0.1744 ± 0.0011 |
| vs-2-2 | 21 | 30470 ± 180 | 1.35 ± 0.02 | 194900 ± 440 | 1.54 ± 0.01 | 0.1563 ± 0.001 |
| vs-2-3 | 27 | 30660 ± 180 | 1.36 ± 0.02 | 179200 ± 430 | 1.48 ± 0.01 | 0.1711 ± 0.0011 |
| vs-2-4 | 36 | 27270 ± 170 | 1.36 ± 0.02 | 201750 ± 450 | 1.37 ± 0.01 | 0.1352 ± 0.0009 |
| vs-2-5 | 44 | 17030 ± 140 | 1.33 ± 0.02 | 109750 ± 330 | 1.4 ± 0.01 | 0.1552 ± 0.0013 |
| vs-2-6 | 51 | 23380 ± 160 | 1.33 ± 0.02 | 150540 ± 390 | 1.43 ± 0.01 | 0.1553 ± 0.0011 |
| vs-2-7 | 59 | 16240 ± 130 | 1.37 ± 0.02 | 110340 ± 330 | 1.4 ± 0.01 | 0.1471 ± 0.0013 |
| vs-2-8 | 69 | 28090 ± 170 | 1.35 ± 0.02 | 167180 ± 410 | 1.44 ± 0.01 | 0.168 ± 0.0011 |
| vs-2-9 | 73 | 35960 ± 190 | 1.44 ± 0.02 | 191070 ± 440 | 1.61 ± 0.01 | 0.1882 ± 0.0011 |
| vs-2-10 | 82 | 38160 ± 200 | 1.32 ± 0.01 | 310760 ± 560 | 1.63 ± 0.01 | 0.1228 ± 0.0007 |
| vs-2-11 | 90 | 178290 ± 430 | 1.39 ± 0.01 | 783240 ± 890 | 1.7 ± 0.01 | 0.2276 ± 0.0006 |
| vs-2-12 | 99 | 436900 ± 660 | 1.48 ± 0.01 | 1673510 ± 1300 | 1.79 ± 0.01 | 0.2611 ± 0.0004 |
| vs-2-13 | 108 | 558350 ± 750 | 1.43 ± 0.01 | 2314240 ± 1530 | 1.71 ± 0.01 | 0.2413 ± 0.0004 |
| vs-2-14 | 115 | 2068180 ± 1440 | 1.46 ± 0.01 | 7446540 ± 2740 | 1.84 ± 0.01 | 0.2777 ± 0.0002 |
| vs-2-15 | 123 | 1779180 ± 1340 | 1.44 ± 0.01 | 7234510 ± 2700 | 1.73 ± 0.01 | 0.2459 ± 0.0002 |
| vs-2-16 | 128 | 2497380 ± 1590 | 1.49 ± 0.01 | 9267760 ± 3050 | 1.85 ± 0.01 | 0.2695 ± 0.0002 |
|  | | | | | | |
| vs-3-2 | 16 | 180040 ± 430 | 1.40 ± 0.01 | 774670 ± 880 | 1.6 ± 0.01 | 0.2324 ± 0.0006 |
| vs-3-3 | 24 | 245380 ± 500 | 1.43 ± 0.01 | 1012100 ± 1010 | 1.68 ± 0.01 | 0.2424 ± 0.0005 |
| vs-3-4 | 32 | 381170 ± 620 | 1.44 ± 0.01 | 1527940 ± 1240 | 1.7 ± 0.01 | 0.2495 ± 0.0005 |
| vs-3-5 | 38 | 278470 ± 530 | 1.38 ± 0.01 | 1219320 ± 1110 | 1.63 ± 0.01 | 0.2284 ± 0.0005 |
| vs-3-6 | 44 | 136350 ± 370 | 1.45 ± 0.01 | 605110 ± 780 | 1.72 ± 0.01 | 0.2253 ± 0.0007 |
| vs-3-7 | 52 | 119180 ± 350 | 1.42 ± 0.01 | 557280 ± 750 | 1.67 ± 0.01 | 0.2139 ± 0.0007 |
| vs-3-8 | 58 | 93780 ± 310 | 1.40 ± 0.01 | 479230 ± 700 | 1.54 ± 0.01 | 0.1957 ± 0.0007 |
| vs-3-9 | 196 | 3390720 ± 1850 | 1.45 ± 0.01 | 12624090 ± 3570 | 1.85 ± 0.01 | 0.2686 ± 0.0002 |
| vs-3-10 | 201 | 2980400 ± 1730 | 1.43 ± 0.01 | 12307810 ± 3520 | 1.83 ± 0.01 | 0.2422 ± 0.0002 |
| vs-3-11 | 206 | 2739760 ± 1660 | 1.44 ± 0.01 | 10511600 ± 3250 | 1.79 ± 0.01 | 0.2606 ± 0.0002 |
| vs-3-12 | 211 | 1703270 ± 1310 | 1.49 ± 0.01 | 5401880 ± 2330 | 1.73 ± 0.01 | 0.3153 ± 0.0003 |
|  | | | | | | |
| vs-4-2 | 26 | 30170 ± 180 | 1.41 ± 0.02 | 163030 ± 410 | 1.6 ± 0.01 | 0.185 ± 0.0012 |
| vs-4-3 | 32 | 23820 ± 160 | 1.34 ± 0.02 | 142360 ± 380 | 1.49 ± 0.01 | 0.1673 ± 0.0012 |
| vs-4-4 | 40 | 39220 ± 200 | 1.34 ± 0.01 | 217850 ± 470 | 1.52 ± 0.01 | 0.18 ± 0.001 |
| vs-4-5 | 46 | 56710 ± 240 | 1.42 ± 0.01 | 335470 ± 580 | 1.7 ± 0.01 | 0.1691 ± 0.0008 |
| vs-4-6 | 53 | 65290 ± 260 | 1.38 ± 0.01 | 339860 ± 590 | 1.49 ± 0.01 | 0.1921 ± 0.0008 |
| vs-4-7 | 59 | 67530 ± 260 | 1.35 ± 0.01 | 368140 ± 610 | 1.51 ± 0.01 | 0.1834 ± 0.0008 |
| vs-4-8 | 69 | 123410 ± 350 | 1.38 ± 0.01 | 559950 ± 750 | 1.6 ± 0.01 | 0.2204 ± 0.0007 |
| vs-4-9 | 72 | 158330 ± 400 | 1.42 ± 0.01 | 652120 ± 810 | 1.57 ± 0.01 | 0.2428 ± 0.0007 |
| vs-4-10 | 78 | 351070 ± 600 | 1.41 ± 0.01 | 1460540 ± 1210 | 1.7 ± 0.01 | 0.2404 ± 0.0005 |
| vs-4-11 | 86 | 438060 ± 670 | 1.39 ± 0.01 | 1798110 ± 1350 | 1.68 ± 0.01 | 0.2436 ± 0.0004 |
| vs-4-12 | 95 | 2330940 ± 1530 | 1.53 ± 0.01 | 7214910 ± 2700 | 1.89 ± 0.01 | 0.3231 ± 0.0002 |
|  | | | | | | |
| vs-5-1 | 56 | 525450 ± 730 | 1.35 ± 0.01 | 2801640 ± 1680 | 1.61 ± 0.01 | 0.1876 ± 0.0003 |
| vs-5-2 | 62 | 576440 ± 760 | 1.42 ± 0.01 | 2538900 ± 1600 | 1.71 ± 0.01 | 0.227 ± 0.0003 |
| vs-5-3 | 71 | 1149090 ± 1080 | 1.47 ± 0.01 | 4523200 ± 2130 | 1.81 ± 0.01 | 0.254 ± 0.0003 |
| vs-5-4 | 78 | 761350 ± 880 | 1.42 ± 0.01 | 3238670 ± 1810 | 1.72 ± 0.01 | 0.2351 ± 0.0003 |
| vs-5-5 | 86 | 1899580 ± 1380 | 1.45 ± 0.01 | 6796840 ± 2620 | 1.8 ± 0.01 | 0.2795 ± 0.0002 |
| vs-5-6 | 93 | 2273440 ± 1510 | 1.44 ± 0.01 | 8754040 ± 2970 | 1.79 ± 0.01 | 0.2597 ± 0.0002 |
| vs-5-7 | 99 | 1363920 ± 1170 | 1.46 ± 0.01 | 5451870 ± 2340 | 1.85 ± 0.01 | 0.2502 ± 0.0002 |
| vs-5-8 | 106 | 1328730 ± 1160 | 1.45 ± 0.01 | 5174900 ± 2280 | 1.81 ± 0.01 | 0.2568 ± 0.0003 |
| vs-5-9 | 38 | 893760 ± 950 | 1.41 ± 0.01 | 3594520 ± 1900 | 1.73 ± 0.01 | 0.2486 ± 0.0003 |
| vs-5-10 | 59 | 731420 ± 860 | 1.38 ± 0.01 | 3359900 ± 1840 | 1.64 ± 0.01 | 0.2177 ± 0.0003 |
| vs-5-11 | 66 | 1305010 ± 1150 | 1.42 ± 0.01 | 5313050 ± 2310 | 1.76 ± 0.01 | 0.2456 ± 0.0002 |

***Stage 2: apparent dose profiling and selecting sampled for further analysis***

Mineral preparation procedures similar to those used by Burbidge et al. (2007) and Kinnaird et al. (2017) were used to extract HF-etched ‘quartz’ from each of the profiled samples. Paired aliquots of ‘quartz’ were subjected to a simplified four step SAR procedure (with repeat and zero doses) to obtain estimates of stored dose (Gy) and sensitivity (counts per Gy), and assess apparent dose and sensitivity distributions (Fig. S2).

**Figure S.2** *–* the methodological approach of Turner et al. (2021) and Kinnaird et al. (2017): from *stage 1*, field-based luminescence screening, through *stage 2,* calibrated luminescence characterisation, to *stage 3,* OSL dating

***
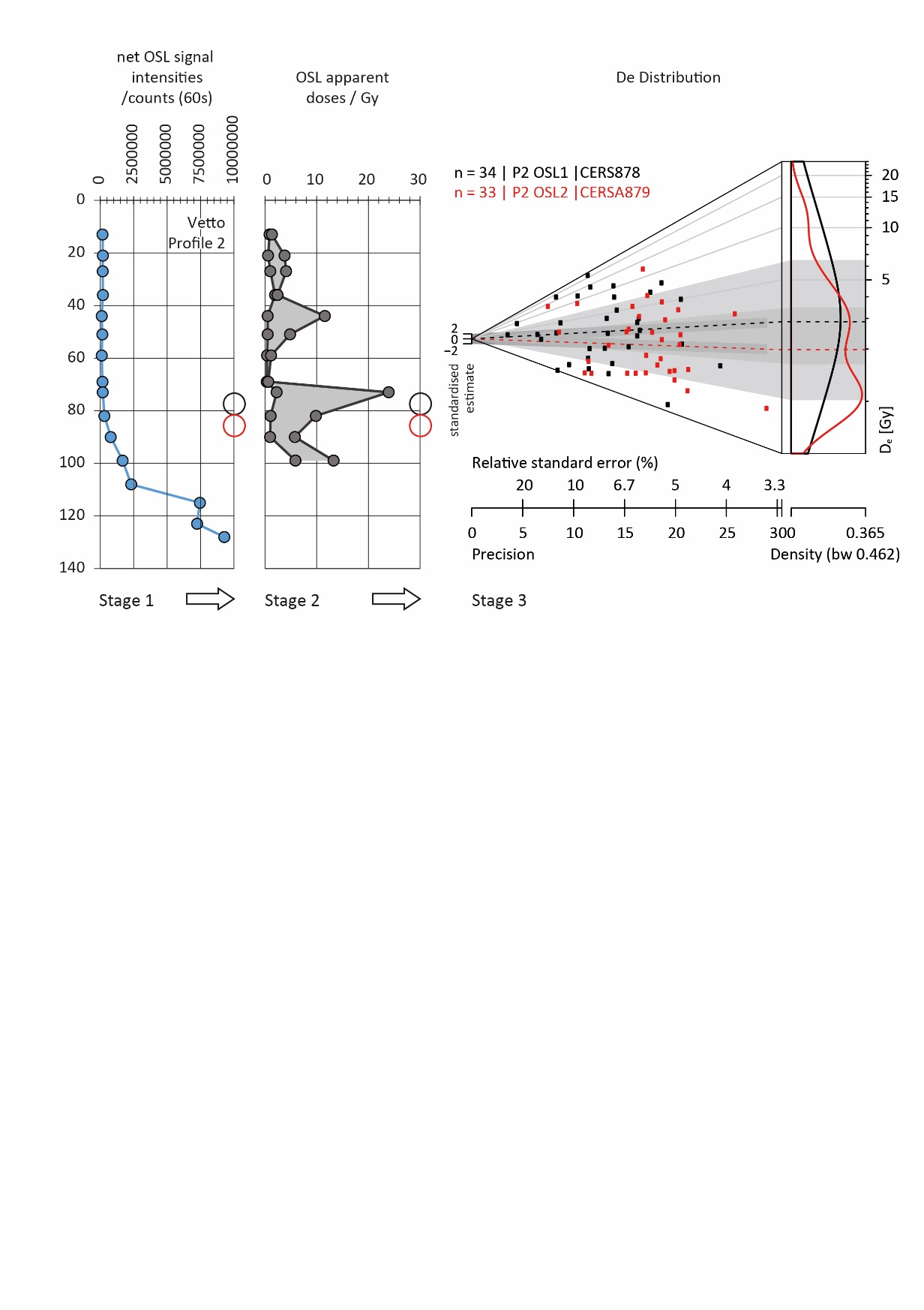
***

***Stage 3: constructing sediment chronologies and defining the constructional sequence to the Vetto terrace walls and earthworks***

A luminescence age is the quotient of the burial dose (quantified as equivalent dose, in Gy) over the effective total environmental dose rate (in Gy.ka^-1^).

*3a. Equivalent dose (D_e_) determination.* Standard mineral preparations procedures were used to extract sand-sized quartz from each sample: all samples were wet-sieved to extract the 90-250 μm fractions, which were then treated in hydrochloric acid (HCl) and hydrogen peroxide (H_2_O_2_), to remove carbonate and organic matter respectively (Srivastava et al., 2019; Kinnaird et al., 2017). Quartz was then concentrated using LST heavy liquid (lithium heteropolytungstates in water) density separation. The quartz concentrates were then etched in hydrofluoric acid (HF), followed by a treatment in HCl to remove fluoride precipitates. These prepared quartz fractions were re-sieved at 150 μm, and the 90-150 μm sized fractions, dispensed to disc for equivalent dose determinations.

All OSL measurements were carried out using a Risø TL/OSL DA-20 automated dating system, equipped with a ^90^Sr/^90^Y β-source for irradiation, blue LEDs emitting around 470nm and infrared diodes emitting around 830nm for optical stimulation. OSL was detected through 7.5mm of Huoya U-340 filter and detected with a 9635QA photomultiplier tube. Des were determined using a SAR protocol (Murray and Wintle, 2000) with a pre-heat of 220°C for 10 s and OSL measurement at 125°C for 60 s. The protocol comprised three to four regenerative doses, with additional cycles for zero dose, a repeat recycling dose and an IRSL dose. Luminescence signals were screened using standardised rejection criteria, and aliquots satisfying the following criteria were accepted for final D_e_ calculation: 1) recuperation of less than 5 %; 2) recycling ratio within 10 % of unity, including uncertainties (Murray and Wintle, 2003); 3) OSL IR depletion ratio within 10 % of unity (Duller, 2003) and 4) test dose signals 3σ greater than background levels. To generate a D_e_, logged minimum dose model (MDM; Galbraith et al., 1999) was used.

*3b. Dose rate determinations.* The calculation of the total environmental dose rate (Ḋ) can be summarised by:

Ḋ = Ḋ_α_ + Ḋ_β_ + Ḋ_γ_ + Ḋ_cosmic_

where Ḋ_α_ = environmental dose from alpha radiation, which can be internal and external to the grain, Ḋ_β_ = beta dose rate, again internal and external, Ḋ_γ_ = gamma dose rate, external and Ḋ_cosmic_ = cosmic dose rate. As the quartz concentrates had been etched in HF to remove the alpha irradiated outer layer of the grains, calculation of Ḋ_α_ is not required. Ḋ_β_ and Ḋ_γ_ were calculated from radionuclide concentrations (^232^Th, ^238^U and ^40^K) measured using Inductively Coupled Plasma Mass Spectrometry/Optical Emission Spectrometry at X-Ray Mineral Services Ltd., UK. These radionuclide concentrations were converted into infinite matrix Ḋs using the conversion factors of Guerin et al. (2011). Infinite matrix Ḋs require adjustments, including grain size, etching and water content. The range of beta particles may be comparable to the diameter of grains under investigation, thus Ḋ_β_ is corrected for grain size attenuation effects, using the attenuation factors of Guerin et al. (2012). Secondary attenuation caused by chemical etching of quartz grains was adjusted using Bell’s (1979) attenuation factors. Ḋ_γ_ does not require any grain size related attenuation correction, however, moisture in the sediment matrix attenuates both Ḋ_β_s and Ḋ_γ_s which would otherwise reach the grain and contribute to the total Ḋ, and hence were corrected for. Ḋ_cosmic_ was calculated following the calculations of Prescott and Hutton (1994). All Ḋs calculations were made using the DRAC (v1.2) software of Durcan et al. (2015), an open access, web-based program which enables Ḋ calculation for trapped charge dating applications. Dose rates to the prepared quartz fraction are summarised in Table S.2.

*3c. Results*. The results from stages 3a and 3b are tabulated in Tables S.2 to S.4. The samples show similar potassium (K), uranium (U) and thorium (Th) concentrations: K varying around 1.6. ± 0.1 %, U, around 0.8 ± 0.3 ppm and Thorium, 8.8 ± 0.3 ppm. These concentrations contribute to an effective dose rate to the HF-etched quartz of approximately 2.46 ± 0.08 Gy ka^-1^.

**Table S.2** *- Summary of the status of OSL dating samples included in the report.*

| **Area** | **Field Code** | **Equivalent to** | **Depth (cm)** | **Lab Code** | **Notes** |
| --- | --- | --- | --- | --- | --- |
| Vetto East | P1 OSL1 | *VS/1-5 to 6* | 90 | CERSA876 | Minimum age reported |
|  | P1 OSL2 | *VS/1-13 to 14* | 55 | CERSA877 |  |
|  | P2 OSL1 | *VS/2-9 to 10* | 78 | CERSA878 | Absolute date reported |
|  | P2 OSL2 | *VS/2-10 to 11* | 86 | CERSA879 |  |
|  | P3 OSL1 | *VS/3-7 to 8* | 55 | CERSA880 |  |
|  | P4 OSL1 | *VS/4-7* | 62 | CERSA881 |  |
|  | P4 OSL2 | *VS/4-10* | 79 | CERSA882 |  |
|  | P5 OSL1 | *VS/5-10 to 11* | 63 | CERSA883 |  |

**Table S.3** *- Radionuclide concentrations, beta, gamma, cosmic and the total dose rates for all samples in this report, with all calculations made prior to rounding.*

| **Lab code** | **Th (ppm)** | **U (ppm)** | **K (%)** | **Ḋ_β_ (Gy.ka^-1^)** | **Ḋ_γ_ (Gy.ka^-1^)** | **Ḋ_cosmic_ (Gy.ka^-1^)** | **Total Ḋ (Gy.ka^-1^)** |
| --- | --- | --- | --- | --- | --- | --- | --- |
| 878 | 8.35 ± 0.83 | 2.24 ± 0.22 | 1.59 ± 0.16 | 1.34 ± 0.10 | 0.86 ± 0.05 | 0.19 ± 0.02 | 2.39 ± 0.12 |
| 879 | 8.58 ± 0.86 | 2.23 ± 0.22 | 1.58 ± 0.16 | 1.42 ± 0.11 | 0.91 ± 0.05 | 0.19 ± 0.02 | 2.51 ± 0.12 |
| 880 | 8.66 ± 0.87 | 2.37 ± 0.24 | 1.59 ± 0.16 | 1.30 ± 0.10 | 0.84 ± 0.05 | 0.21 ± 0.02 | 2.35 ± 0.11 |
| 881 | 8.80 ± 0.88 | 2.36 ± 0.24 | 1.61 ± 0.16 | 1.40 ± 0.11 | 0.90 ± 0.05 | 0.20 ± 0.02 | 2.50 ± 0.12 |
| 882 | 8.95 ± 0.89 | 2.42 ± 0.24 | 1.61 ± 0.16 | 1.34 ± 0.10 | 0.88 ± 0.05 | 0.19 ± 0.02 | 2.41 ± 0.12 |
| 883 | 8.95 ± 0.89 | 2.48 ± 0.25 | 1.54 ± 0.15 | 1.32 ± 0.10 | 0.88 ± 0.05 | 0.20 ± 0.02 | 2.40 ± 0.11 |

Individual depositional ages range between 0.28 ± 0.05 ka (CERSA 878) and 1.17 ± 0.20 ka (CERSA 883), or CE1750 and 860, respectively. Age uncertainties vary between 10 and 22 %, with the younger samples characterised by larger uncertainties, due to the over-dispersed dose distributions, and lower precision due to less bright signals. Two samples, CERSA 876 and 877, yielded D_e_ values of >28 Gy and >38 Gy. Assuming a dose rate of 2.46 ± 0.08 Gy.ka^-1^, the samples result in minimum ages of >11.4 and >15.4 ka.

**Table S.4** *- Summary OSL data. De, Ḋ, and ages are shown to two decimal places, with all calculations made prior to rounding. All dates are relative to the year 2022.*

| **Field code** | **Lab code** | **Depth (cm)** | **MDM D_e_ (Gy)** | **Total DR (Gy.ka^-1^)** | **Age (ka)** | **Calendar years** |
| --- | --- | --- | --- | --- | --- | --- |
| *P2 OSL1* | *CERSA878* | 78 | 0.66 ± 0.11 | 2.39 ± 0.12 | 0.28 ± 0.05 | CE 1746 ± 48 |
| *P2 OSL2* | *CERSA879* | 86 | 0.94 ± 0.13 | 2.51 ± 0.12 | 0.37 ± 0.06 | CE 1648 ± 55 |
| *P3 OSL1* | *CERSA880* | 55 | 2.34 ± 0.29 | 2.35 ± 0.11 | 0.99 ± 0.13 | CE 1026 ± 133 |
| *P4 OSL1* | *CERSA881* | 62 | 1.41 ± 0.13 | 2.50 ± 0.12 | 0.57 ± 0.06 | CE 1457 ± 59 |
| *P4 OSL2* | *CERSA882* | 79 | 1.63 ± 0.36 | 2.41 ± 0.12 | 0.68 ± 0.15 | CE 1346 ± 153 |
| *P5 OSL1* | *CERSA883* | 63 | 2.80 ± 0.46 | 2.40 ± 0.11 | 1.17 ± 0.20 | CE 857 ± 199 |

**References**

Bell, W.T., 1979. Attenuation factors for the absorbed radiation dose in quartz inclusions for thermoluminescence dating. *Ancient TL*, 8(2), p.12.

Burbidge, C.I., Sanderson, D.C.W., Housley, R.A. and Allsworth Jones, P., 2007, Survey of Palaeolithic sites by luminescence profiling, a case study from Eastern Europe. *Quaternary Geochronology*, 2(1), pp. 296-302.

Duller, G.A.T., 2003. Distinguishing quartz and feldspar in single grain luminescence measurements. *Radiation measurements*, 37(2), pp.161-165.

Durcan, J.A., King, G.E. and Duller, G.A., 2015. DRAC: Dose Rate and Age Calculator for trapped charge dating. *Quaternary Geochronology*, 28, pp.54-61.

Galbraith, R.F., Roberts, R.G., Laslett, G.M., Yoshida, H. and Olley, J.M., 1999. Optical dating of single and multiple grains of quartz from Jinmium rock shelter, northern Australia: Part I, experimental design and statistical models. *Archaeometry*, 41(2), pp.339-364.

Guérin, G., Mercier, N. and Adamiec, G., 2011. Dose-rate conversion factors: update. *Ancient TL*, 29(1), pp.5-8.

Guérin, G., Mercier, N., Nathan, R., Adamiec, G. and Lefrais, Y., 2012. On the use of the infinite matrix assumption and associated concepts: a critical review. *Radiation Measurements*, 47(9), pp.778-785.

Kinnaird, T., Bolos, J., Turner, A. and Turner, S., 2017. Optically-stimulated luminescence profiling and dating of historic agricultural terraces in Catalonia (Spain). *Journal of Archaeological Science*, 78, pp.66-77.

Munyikwa, K., Kinnaird, T.C. and Sanderson, D.C., 2021. The potential of portable luminescence readers in geomorphological investigations: a review. *Earth Surface Processes and Landforms,* 46(1), pp.131-150.

Murray, A.S. and Wintle, A.G., 2000. Luminescence dating of quartz using an improved single-aliquot regenerative-dose protocol. *Radiation measurements*, 32(1), pp.57-73.

Murray, A.S. and Wintle, A.G., 2003. The single aliquot regenerative dose protocol: potential for improvements in reliability. *Radiation measurements*, 37(4-5), pp.377-381.

Prescott, J.R. and Hutton, J.T., 1994. Cosmic ray contributions to dose rates for luminescence and ESR dating: large depths and long-term time variations. *Radiation measurements*, 23(2-3), pp.497-500.

Sanderson, D.C. and Murphy, S., 2010. Using simple portable OSL measurements and laboratory characterisation to help understand complex and heterogeneous sediment sequences for luminescence dating. *Quaternary Geochronology*, 5(2-3), pp.299-305.

Srivastava, A., Durcan, J.A. and Thomas, D.S.G., 2019. Analysis of late quaternary linear dune development in the Thar Desert, India. *Geomorphology*, 344, pp.90-98.

Turner, S., Kinnaird, T., Varinlioğlu, G., Şerifoğlu, T.E., Koparal, E., Demirciler, V., Athanasoulis, D., Ødegård, K., Crow, J., Jackson, M. and Bolòs, J., 2021. Agricultural terraces in the Mediterranean: medieval intensification revealed by OSL profiling and dating. *Antiquity*, 95(381), pp.773-79

## 2. HLC sources

| **Name** | **Publication** | **Type** | **Scale** | **Source** | **HLC Period** |
| --- | --- | --- | --- | --- | --- |
| **Google© Satellite** | 2020 | Satellite Images | - | QuickMapServices plugin [(NextGis 2019)](https://paperpile.com/c/JWXlUf/Ui7fG) in QGIS 3.16-Hannover [(QGIS Development Team 2021)](https://paperpile.com/c/JWXlUf/5uNtO) | 2010s |
| **Bing© Satellite** | 2020 | Satellite Images | - | QuickMapServices plugin [(NextGis 2019)](https://paperpile.com/c/JWXlUf/Ui7fG) in QGIS 3.16-Hannover [(QGIS Development Team 2021)](https://paperpile.com/c/JWXlUf/5uNtO) |  |
| **Carta Tecnica Regionale (CTR)** | 2018 | Cadastral Map | 1:5.000 | WMS service [(“CTR” 2018)](https://paperpile.com/c/JWXlUf/7DSDg) |  |
| **Compagnia Generale Riprese (CGR) Aeree** | 2018 | Aerial Photos | - | WMS service [(“CGR” 2018)](https://paperpile.com/c/JWXlUf/7bsGx) |  |
| **AGEA (Agenzia per le Erogazioni in Agricoltura) 11** | 2011 | Aerial Photos | - | WMS service [(“AGEA” 2011)](https://paperpile.com/c/JWXlUf/kEgwg) |  |
| **AGEA (Agenzia per le Erogazioni in Agricoltura) 08** | 2008 | Aerial Photos | - | WMS service [(“AGEA” 2008)](https://paperpile.com/c/JWXlUf/ErzSd) | 2000s |
| **Volo Compagnia Generale Riprese Aeree (CGRA)** | 1976 - 1978 | Aerial Photos | 1:13.500 | Photos retrieved at the Ufficio cartografico della Provincia di Reggio Emilia [(“CGRA” 1976)](https://paperpile.com/c/JWXlUf/FpjP2) | 1970s |
| **KH-9 (Hexagon)** | 1974 | Satellite Images | - | Declassified image retrieved at the U.S. Geological Survey website [(“USGS EROS Archive - Declassified Data - Declassified Satellite Imagery - 3” n.d.)](https://paperpile.com/c/JWXlUf/JCWyM) |  |
| **Volo GAI (Gruppo Aereo Italiano )** | 1954 - 1955 | Aerial Photos | 1:33.000 | Photos retrieved at the Istituto Geografico Militare (IGM) website [(“IGM” 1954)](https://paperpile.com/c/JWXlUf/WyZy0) | 1950s |
| **Nuovo Catasto Terreni** | 1886 - 1900 | Cadastral Map | 1:2.000 | Map retrieved at the Ufficio cartografico della Provincia di Reggio Emilia [(“Nuovo Catasto Terreni” 1900)](https://paperpile.com/c/JWXlUf/rCw6R) | 19th Century |
| **Carta Storica Regionale Emilia Romagna** | 1853 | Historical Map | 1:50.000 | WMS service [(“Carta Storica Regionale” 1853)](https://paperpile.com/c/JWXlUf/4bizg) |  |
| **Second military survey of the Habsburg Empire** | 1818 - 1829 | Historical Map | 1:28800 | Map retrieved at the Mapire website [(Timár et al. 2006; Ostafin, Pietrzak, and Kaim 2021)](https://paperpile.com/c/JWXlUf/CJW5I+Sl1sA) |  |

For a detailed description of the sources employed to develop the HLC map of the area refer to: *Brandolini, F. & Turner, S. Revealing patterns and connections in the historic landscape of the northern Apennines (Vetto, Italy). Journal of Maps, 1–11 (2022)*.

DOI: [10.1080/17445647.2022.2088305](http://dx.doi.org/10.1080/17445647.2022.2088305)

**References**

AGEA. (2011). Agenzia per le Erogazioni in Agricoltura, 2011

<https://geoportale.regione.emilia-romagna.it/catalogo/dati-cartografici/cartografia-di-base/immagini/layer-2>

Carta Storica Regionale. (1853). <https://geoportale.regione.emilia-romagna.it/catalogo/dati-cartografici/cartografia-di-base/cartografia-storica/layer-1>

CGR. (2018). Compagnia Generale Riprese Aeree, 2018 <https://geoportale.regione.emilia-romagna.it/catalogo/dati-cartografici/cartografia-di-base/immagini/layer-4>

CGRA. (1976). Volo Compagnia Generale Riprese Aeree, Provincia Di Reggio Emilia. 1976.

<https://www.provincia.re.it/aree-tematiche/pianificazione-territoriale/sistema-informativo-territoriale/archivio-cartografico/foto-aeree/volo-cgra-1976-78-scala-113-500>/

CTR. (2018). Carta Tecnica Regionale, 2018. <https://geoportale.regione.emilia-romagna.it/catalogo/dati-cartografici/cartografia-di-base/cartografia-tecnica/layer-1>

IGM. (1954). Istituto Geografico Militare. 1954. [https://www.igmi.org](https://www.igmi.org/)/

NextGis. (2019). QuickMapServices (version 0.19.11). <https://github.com/nextgis/quickmapservices>

Nuovo Catasto Terreni. (1900). <https://www.provincia.re.it/aree-tematiche/pianificazione-territoriale/sistema-informativo-territoriale/archivio-cartografico/cartografia-storica/carta-catastale-di-impianto-della-provincia-di-reggio-emilia-scala-12-000-11-000-edizione-fine-xix-secolo>/

Ostafin, K., Pietrzak, M., & Kaim, D. (2021). Impact of the Cartographer’s position and topographic accessibility on the accuracy of historical land use information: Case of the second military survey maps of the Habsburg empire. *ISPRS International Journal of Geo-Information*, *10*(12), 820. <https://doi.org/10.3390/ijgi10120820>

Timár, G., Molnár, G., Székely, B., Biszak, S., Varga, J., & Jankó, A. (2006). *Digitized maps of the Habsburg empire – the map sheets of the second military survey and their georeferenced version*. Arcanum.

USGS EROS Archive – Declassified Data – Declassified Satellite Imagery – 3. (n.d). Retrieved December 13, 2021, from <https://www.usgs.gov/centers/eros/science/usgs-eros-archive-declassified-data-declassified-satellite-imagery-3>
